# Pan-cancer analysis reveals the prognostic and immunotherapeutic value of cytoskeleton-associated protein 2-like

**DOI:** 10.1038/s41598-023-35633-3

**Published:** 2023-05-24

**Authors:** Bocun Yi, Qingfeng Fu, Zhiwen Zheng, Man Zhang, Dongze Liu, Zhengxin Liang, Shengxian Xu, Zhihong Zhang

**Affiliations:** 1 Department of Urology, Tianjin Institute of Urology, The Second Hospital of Tianjin Medical University, Tianjin, China; 2 Tianjin Key Laboratory of Metabolic Diseases, Tianjin Institute of Endocrinology, Chu Hsien-I Memorial Hospital of Tianjin Medical University, Tianjin, China

**Keywords:** Cancer microenvironment, Oncogenes, Tumour biomarkers, Urological cancer

## Abstract

Cytoskeleton-associated protein 2-like (CKAP2L), a cell cycle-related protein, is correlated to tumor progression in some tumors. But there were no pan-cancer studies on CKAP2L, and its role in cancer immunotherapy is also unclear. The expression levels, expression activity, genomic alterations, DNA methylation and functions of CKAP2L in various tumors, as well as the associations between CKAP2L expression and patient prognosis, chemotherapy sensitivity, and tumor immune microenvironment, were all analyzed in a comprehensive pan-cancer analysis of CKAP2L by various databases, analysis websites, and R software. The experiments were also conducted to verify the analysis results. In the majority of cancers, CKAP2L expression and activity were markedly elevated. Elevated CKAP2L expression led to poor prognostic outcomes in patients, and is an independent risk factor for most tumors. Elevated CKAP2L causes decreased sensitivity to chemotherapeutic agents. Knockdown of CKAP2L significantly inhibited the proliferation and metastasis capacity of the KIRC cell lines and resulted in cell cycle G2/M arrest. In addition, CKAP2L was closely related to immune subtypes, immune cell infiltration, immunomodulators and immunotherapy markers (TMB, MSI), patients with high CKAP2L expression were more sensitive to immunotherapy in the IMvigor210 cohort. The results indicate that CKAP2L is a pro-cancer gene that serves as a potential biomarker for predicting patient outcomes. By inducing cells to transition from the G2 phase to the M phase, CKAP2L may promote cell proliferation and metastasis. Furthermore, CKAP2L is closely related to the tumor immune microenvironment and can be used as a biomarker to predict tumor immunotherapy.

## Introduction

Cancer is a major public health issue around the world. Recent data show that by 2022, the United States will have about 5250 cancer cases and 1700 cancer-related deaths every day^1^. One type of cancer that affects many people is renal cell carcinoma (RCC), which accounts for 3.8% of all new cancer cases, of which 70% of these were the renal clear cell carcinoma (KIRC) type, posing a serious threat to human life and health^2^. To overcome cancers, we must continue to improve cancer diagnosis and treatment methods. Of note, pan-cancer analysis uses data from different databases to analyze the relationship between genes and tumor-related factors in different tumor types, thus providing the corresponding biological targets for the diagnosis and treatment of tumors.

The CKAP2L gene is located on chromosome 2 and encodes a mitotic spindle protein thought to be important for neural stem or progenitor cells^3^. It has been suggested that loss-of-function mutations in this gene are the main cause of Filippi's syndrome^4^. So far, CKAP2L has only been studied in a few types of tumors and has been shown to be highly expressed and to promote tumor progression in prostate cancer^5^, glioblastoma^6^, oesophageal squamous cell carcinoma^7^, non-small cell lung cancer^8^ and breast cancer^9^, where it is a potential prognostic biomarker. However, no studies have yet explored CKAP2L at the pan-cancer level.

For many years, cancer research has focused on the malignant cancer cells themselves, neglecting the role of the tumor microenvironment (TME). The TME is a vital mediator of cancer progression and therapeutic effect, and immune cells are important components of the TME^10,11^. It has been shown that all stages of tumors involve complex and dynamic interactions between cancer cells and the immune system, and a rising number of immune cell types have been demonstrated to play a significant role in regulating cancer progression^12^. But until now, there are few studies focused on the relationship between CKAP2L and tumor immunity.

In this study, we explored the expression and activity of CKAP2L in 33 types of tumors and its association with prognosis. We also evaluated its potential impact on the tumor immune microenvironment and immunotherapy response. In the majority of cancers, its expression was positively related to Th2 cells, neutrophils, and myeloid-derived suppressor cells (MDSC). The role of CKAP2L in immunotherapy was also studied. In addition, given that the relationship between CKAP2L and KIRC remains unclear so far, we performed an in-depth analysis of KIRC to fill this gap.

Overall, we have investigated the function of CKAP2L at the pan-cancer level, which may offer evidence for its potential clinical application in the future.

## Results

### The changes of CKAP2L across cancer types

**Figure 1:**
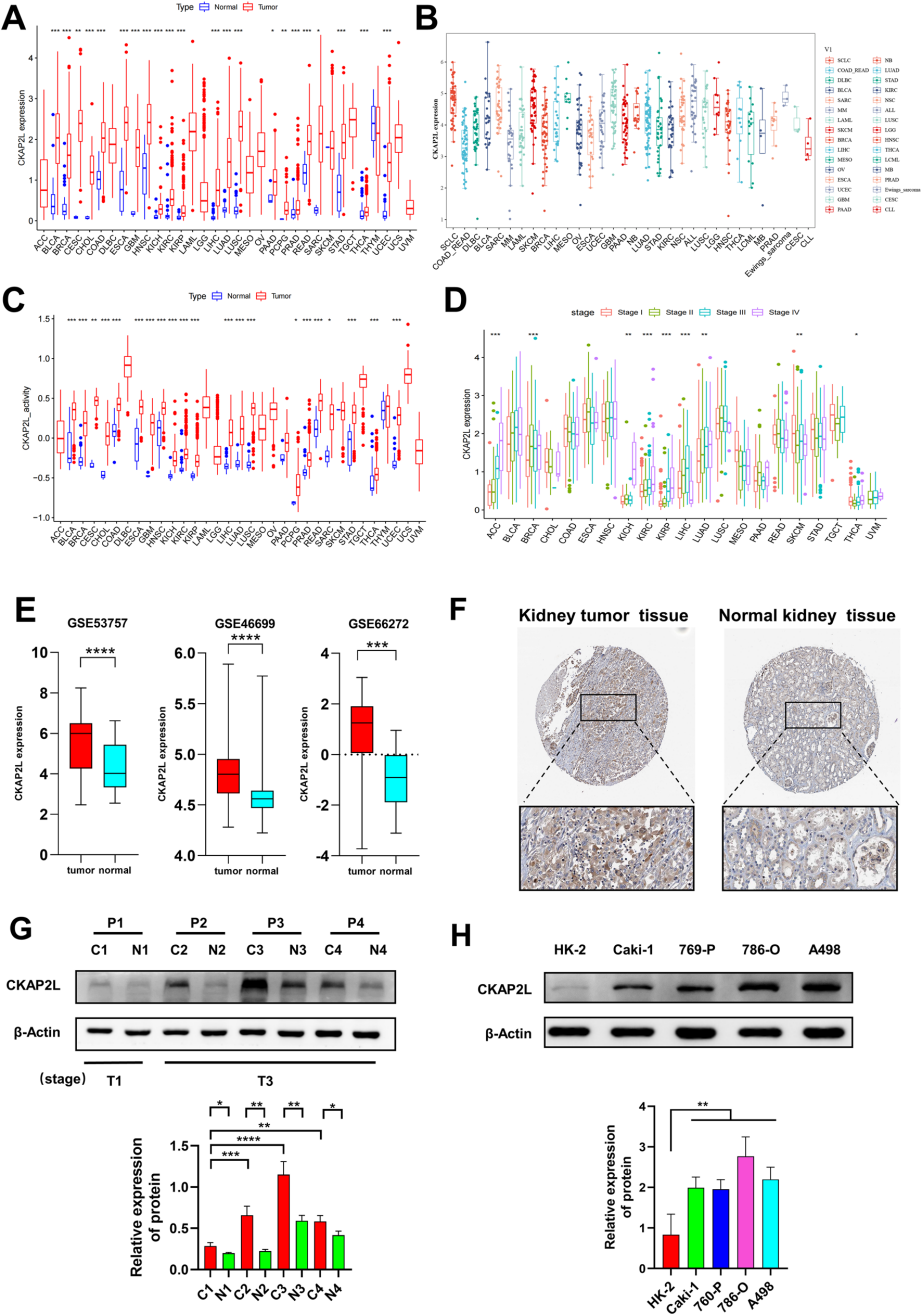
The changes of CKAP2L across cancer types. (*p < 0.05; **p < 0.01; and ***p < 0.001; ****p < 0.0001). (**A**) CKAP2L was differentially expressed in the tumor and normal groups, and its expression was elevated in most tumors. (**B**) Expression of CKAP2L in various cancer cell types according to the CCLE database. (**C**) Differential activity analysis between the tumor and normal groups of CKAP2L. (**D**) Correlation between tumor stage and CKAP2L. (**E**) Differential analysis of CKAP2L expression in KIRC and normal tissues was performed by three cohorts of the GEO database. The expression of CKAP2L was elevated in tumor tissues. (**F**) Immunohistochemical detection of CKAP2L expression data in tumor tissues and normal tissues of KIRC patients were obtained from the HPA website. (**G**) CKAP2L expression levels in tumor tissues and adjacent tissues of four KIRC patients were measured using western-blot assays. CKAP2L expression was higher in tumor tissues compared to adjacent tissues and increased with the TNM stage of patients. Regarding the TMN staging of the four patients, P1 was stage T1, P2, P3 and P4 were stage T3. The original data can be obtained in Supplementary Fig. S5. (**H**) Expression of CKAP2L in HK-2, Caki-1, 769-P, 786-O and A498 cells. The expression level of CKAP2L was higher in tumor cells than in normal renal cells. The original data can be obtained in Supplementary Fig. S6.

The expression levels of CKAP2L in 33 tumors and normal tissues were analyzed by the TCGA database and TIMER2.0, finding that CKAP2L was differentially highly expressed in most cancers. As shown in the figures, there were 22 tumors with elevated CKAP2L expression in the TCGA database (Fig. 1A) and 19 tumors in TIMER2.0 (Supplementary Fig. S1). The CKAP2L expression in various cancer cells obtained through the CCLE database is summarized in Fig. 1B. We observed that CKAP2L expression was high in most tumor cells, which is consistent with the data from TCGA. According to the TCGA database, the activity of CKAP2L was elevated in 21 cancers (Fig. 1C), which was nearly consistent with the results of CKAP2L expression levels. This indicated that these tumors had high levels of CKAP2L expression and activity. As demonstrated in Fig. 1D, CKAP2L expression was related to the tumor stage in some cancers and increased with tumor stages in the ACC, BRCA, KICH, KIRC, KIRP, LUAD and THCA groups. Analysis of genetic alterations showed that the alterations with the highest percentage of CKAP2L in tumors were amplification and mutation (Supplementary Fig. S2A), and these genetic alterations may contribute to the high expression of CKAP2L in tumor tissues. The general profile of genetic alterations in CKAP2L was shown in Supplementary Fig. S2B.

Utilizing three cohorts from the GEO database, we analyzed the transcriptome-level expression of CKAP2L in KIRC and normal tissues. In tumor tissues, CKAP2L expression was significantly higher than in normal tissues (Fig. 1E). At the protein level, as shown in Fig. 1F,G, CKAP2L expression was significantly higher in tumor tissues than in normal tissues of KIRC patients. And the expression of CKAP2L in tumor tissue increases with the TMN stage of the tumor. In addition, cells of KIRC showed higher levels of CKAP2L expression than human kidney normal epithelial cells (Fig. 1H).

**Figure 2:**
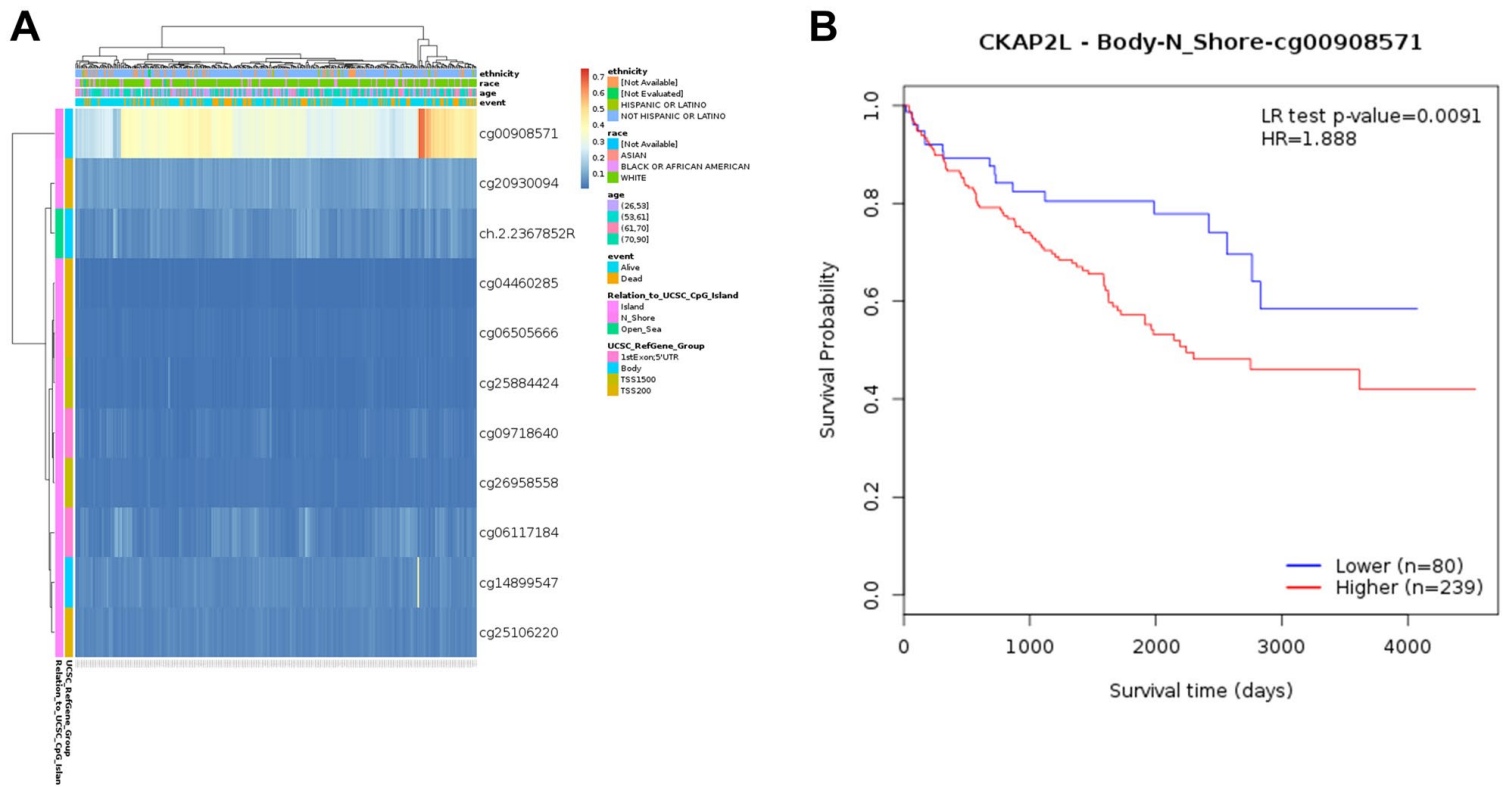
DNA methylation analysis of CKAP2L in KIRC. (**A**) Heat map analysis of DNA methylation expression levels of CKAP2L in KIRC, Heat map methylation levels (1 = fully methylated; 0 = completely unmethylated) are shown as a continuous variable from blue to red. (**B**) Patients with high levels of CKAP2L-cg00908571 methylation had worse OS.

We analyzed CKAP2L methylation levels in KIRC through the MethSurv database and showed its association with clinical features and genetic subregions of patients by the heat map (Fig. 2A). According to the heat map, CKAP2L had the highest methylation at the cg00908571 locus. So, we further investigated the relationship between the level of CKAP2L-cg00908571 methylation and patients' OS. We found that patients with a high level of CKAP2L-cg00908571 methylation possessed a worse overall survival (Fig. 2B).

### Correlation between CKAP2L expression level and prognosis

**Figure 3:**
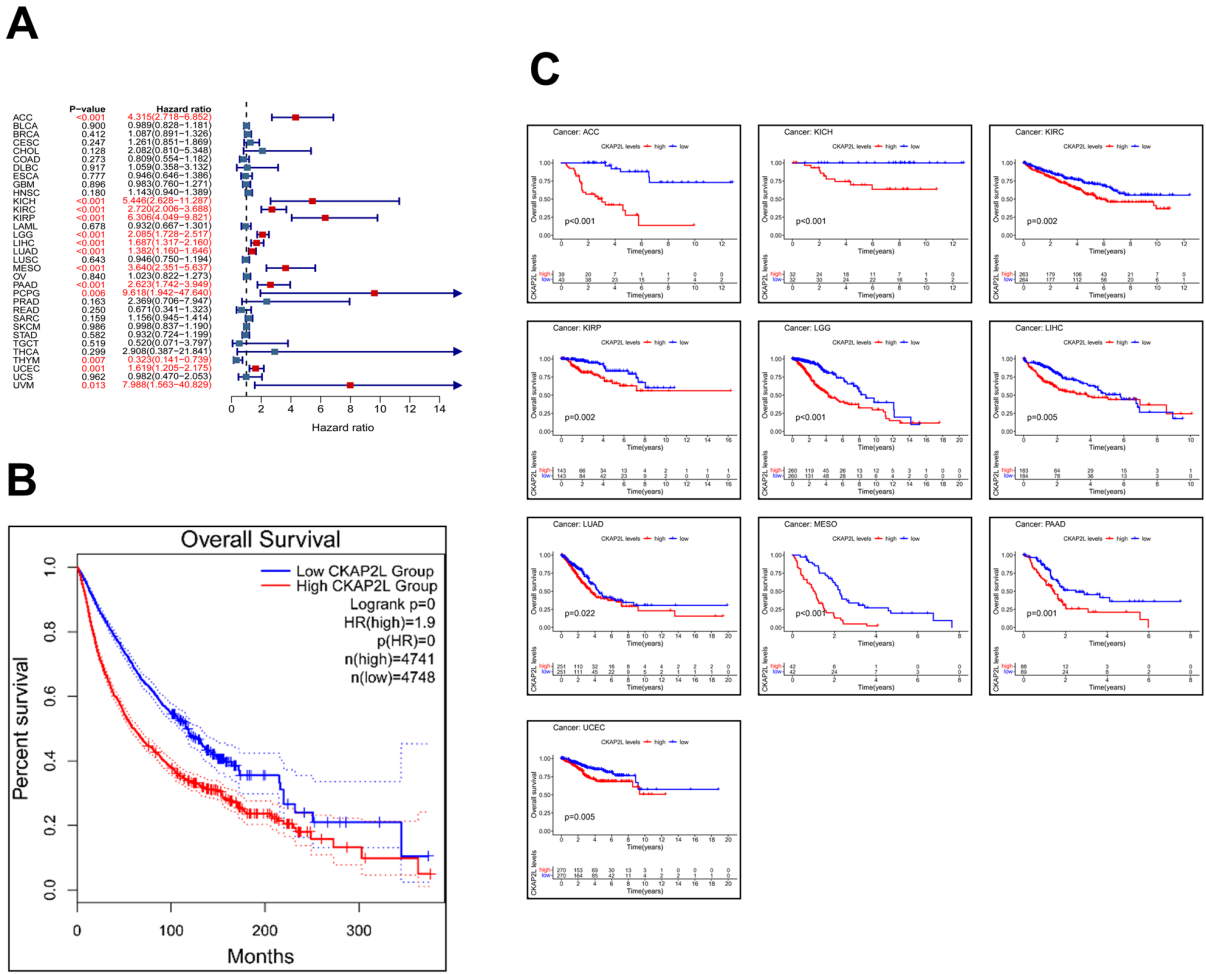
Correlations between CKAP2L expression and OS (the results were displayed with a p-value < 0.05). (**A**) The relationship between CKAP2L expression levels and OS in various cancer types via univariate Cox regression analysis. (**B**) Correlation between OS and CKAP2L expression in total cancer types via KM analysis (HR = 1.9). (**C**) Correlation between OS and CKAP2L expression in each cancer types via KM analysis.

**Figure 4:**
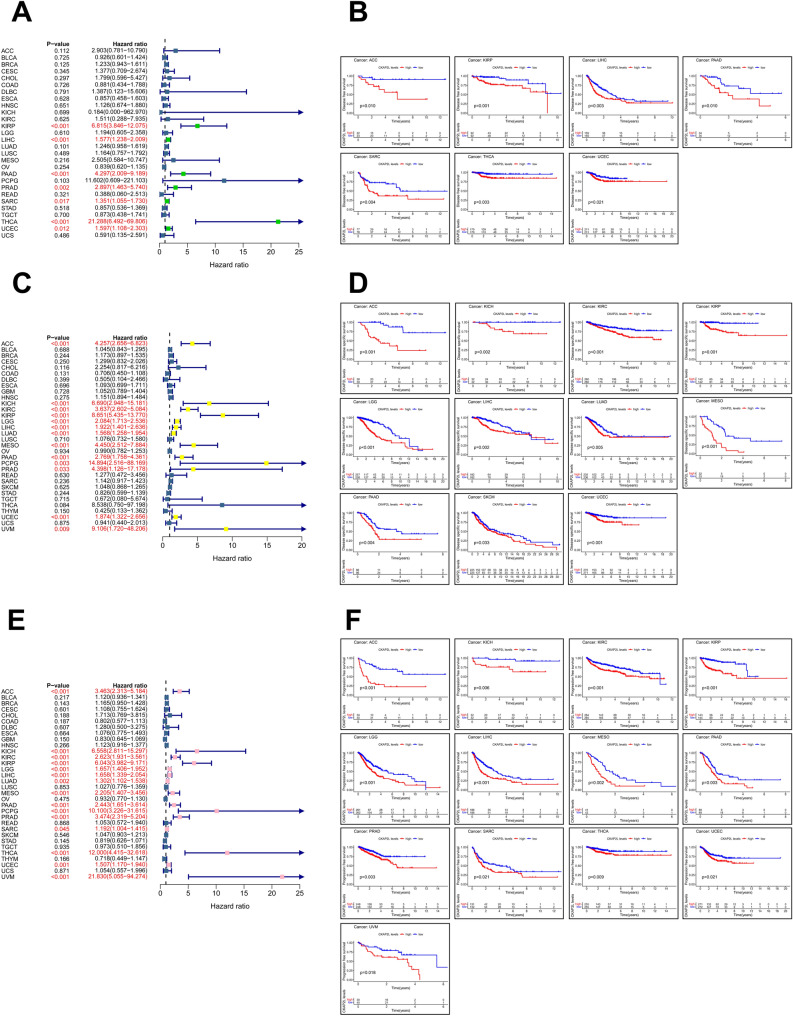
Correlations between CKAP2L expression and DFS, DSS, PFS (the results were displayed with a p-value < 0.05). (**A**) Correlation between CKAP2L expression levels and DFS in total cancer types via univariate Cox regression analysis. (**B**) Correlation between DFS and CKAP2L expression in each cancer types via KM analysis. (**C**) Correlation between CKAP2L expression levels and DSS in total cancers types via univariate Cox regression analysis. (**D**) Correlation between DSS and CKAP2L expression in each cancer types via KM analysis. (**E**) Correlation between CKAP2L expression levels and PFS in total cancer types via univariate Cox regression analysis. (**F**) Correlation between PFS and CKAP2L expression in each cancer types via KM analysis.

At the pan-cancer analysis level, we investigated whether CKAP2L expression is related to patient prognosis using data from the TCGA database. In the forest plots, we can see the results of the univariate Cox analysis. The expression of CKAP2L was significantly related to OS in 13 tumors. Except for THYM, high expression of CKAP2L was related to worse OS in these tumors (Fig. 3A). Regarding DFS, CKAP2L is a risk factor for KIRP, LIHC, PAAD, PRAD, SARC, THCA and UCEC (Fig. 4A). The expression of CKAP2L was related to worse DSS in ACC, KICH, KIRC, KIRP, LGG, LIHC, LUAD, MESO, PAAD, PCPG, PRAD, UCEC and UVM (Fig. 4C). Furthermore, the PFS forest plot showed that CKAP2L is an independent risk factor in the following cancers: ACC, KICH, KIRC, KIRP, LGG, LIHC, LUAD, MESO, PAAD, PCPG, PRAD, SARC, THCA, UCEC and UVM (Fig. 4E).

We also investigated the connection between CKAP2L and prognosis by the Kaplan–Meier algorithm, and the filter condition was p-values < 0.05. According to KM curves, CKAP2L expression was related to worse OS in 10 tumors (Fig. 3C), worse DFS in 7 tumors (Fig. 4B), worse DSS in 11 tumors (Fig. 4D), and worse PFS in 13 tumors (Fig. 4F). Moreover, patients with higher CKAP2L expression had poorer OS in a comprehensive analysis of 33 cancers (Fig. 3B). These data imply that CKAP2L expression is related to a bad prognosis for patients.

### Correlation between CKAP2L expression and chemotherapy drug response

Using the CellMiner database, we attempted to discover a link between the level of CKAP2L expression and the sensitivity of traditional chemotherapeutic medicines. As shown in Supplementary Fig. S3, all drugs except Staurosporine were negatively correlated with the expression of CKAP2L. It suggested that patients with increased CKAP2L may be insensitive to chemotherapeutic drugs.

### Knockdown of CKAP2L expression inhibits KIRC cell proliferation and metastasis

**Figure 5:**
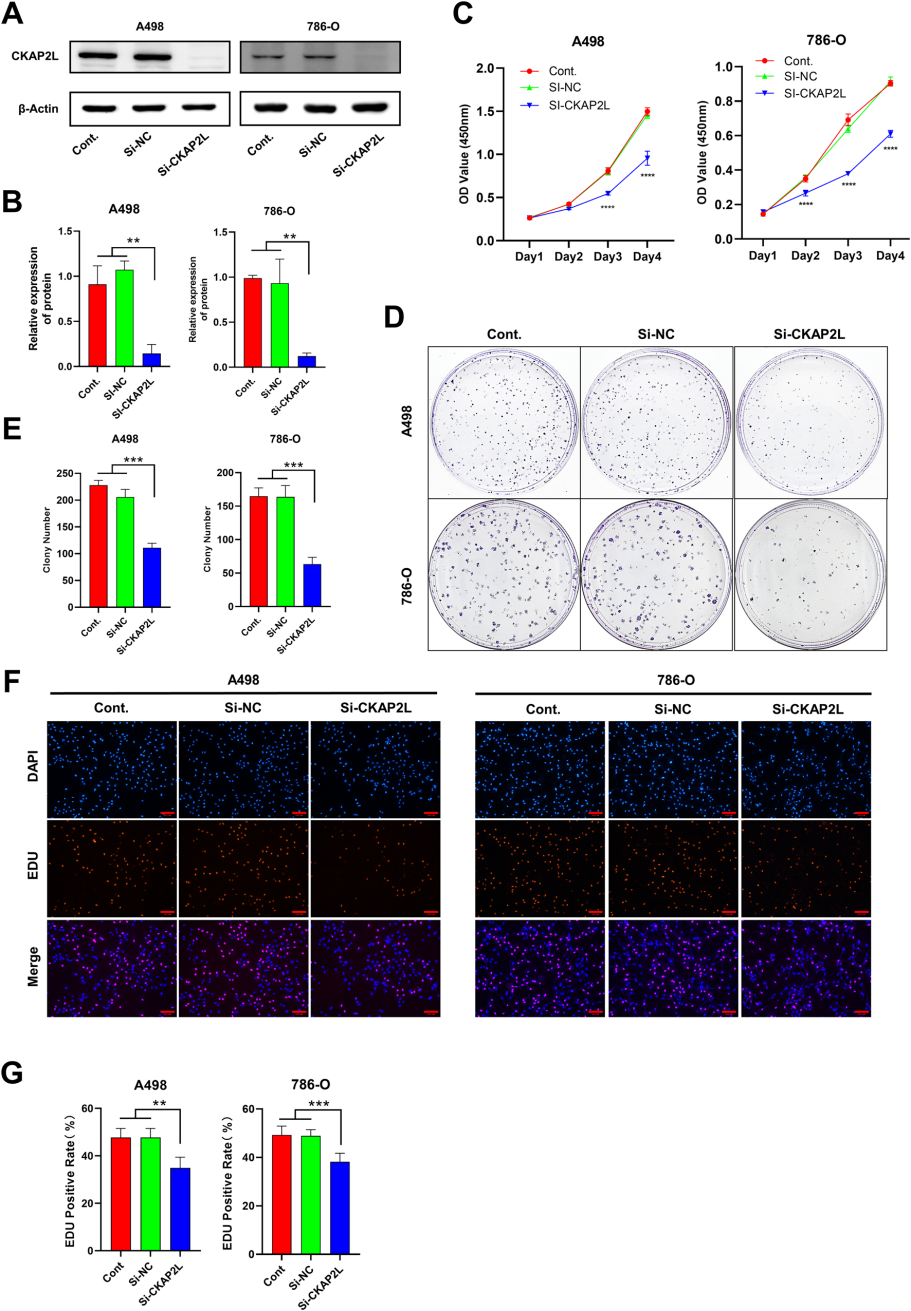
Knockdown of CKAP2L expression inhibits KIRC cell proliferation (scale bar: 500 µm. *p < 0.05; **p < 0.01; ***p < 0.001; ****p < 0.0001). (**A**,**B**) Protein blotting images and quantitative data showed that the expression of CKAP2L in A498 and 786-O cells was significantly reduced after siRNA transfection. The uncropped original image can be obtained in Supplementary Fig. S7. (**C**) CCK8 assay detects the change in the proliferation ability of A498 and 786-O cells after CKAP2L knockdown. (**D**,**E**) Clone formation assay examined the change in the proliferation ability of A498 and 786-O cells after CKAP2L knockdown. (**F**,**G**) EdU incorporation assays assess the change in the proliferation ability of A498 and 786-O cells after CKAP2L knockdown.

**Figure 6:**
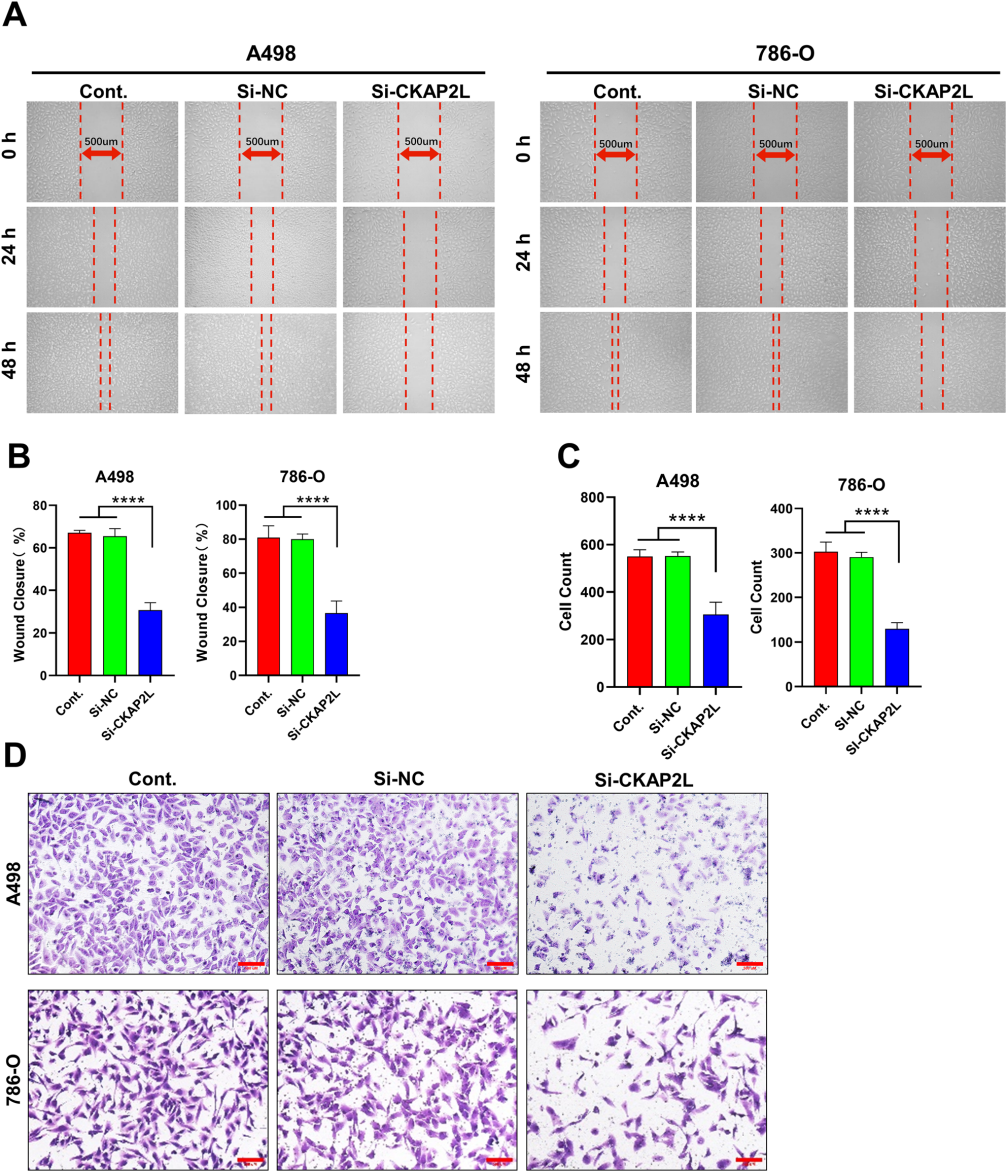
Knockdown of CKAP2L expression suppresses KIRC cell metastasis (scale bar: 500 µm. *p < 0.05; **p < 0.01; ***p < 0.001; ****p < 0.0001). (**A**,**B**) Wound healing assay showed that the migration ability of A498 and 786-O cells was significantly decreased after CKAP2L knockdown. (**C**,**D**) The transwell assay showed that the invasion ability of A498 and 786-O cells was significantly reduced after CKAP2L knockdown.

Because elevated CKAP2L expression is significantly related to worse prognosis in KIRC, we knocked down CKAP2L in A498 cells and 786-O cells to investigate the function of CKAP2L in KIRC. The protein levels of CKAP2L in A498 cells and 786-O cells were significantly decreased after the siRNA transfected (Fig. 5A,B). Next, we performed CCK8, clone formation and EdU incorporation assays to detect changes in cell proliferation capacity. The results (Fig. 5C–G) showed that the deregulation of CKAP2L impaired the proliferation of these cells. Meanwhile, the migration and invasion ability of cells was also significantly reduced with the knockdown of CKAP2L (Fig. 6A–D). These findings suggest that CKAP2L plays a role in cancer cell proliferation and metastasis.

### CKAP2L is involved in the regulation of the cell cycle

**Figure 7:**
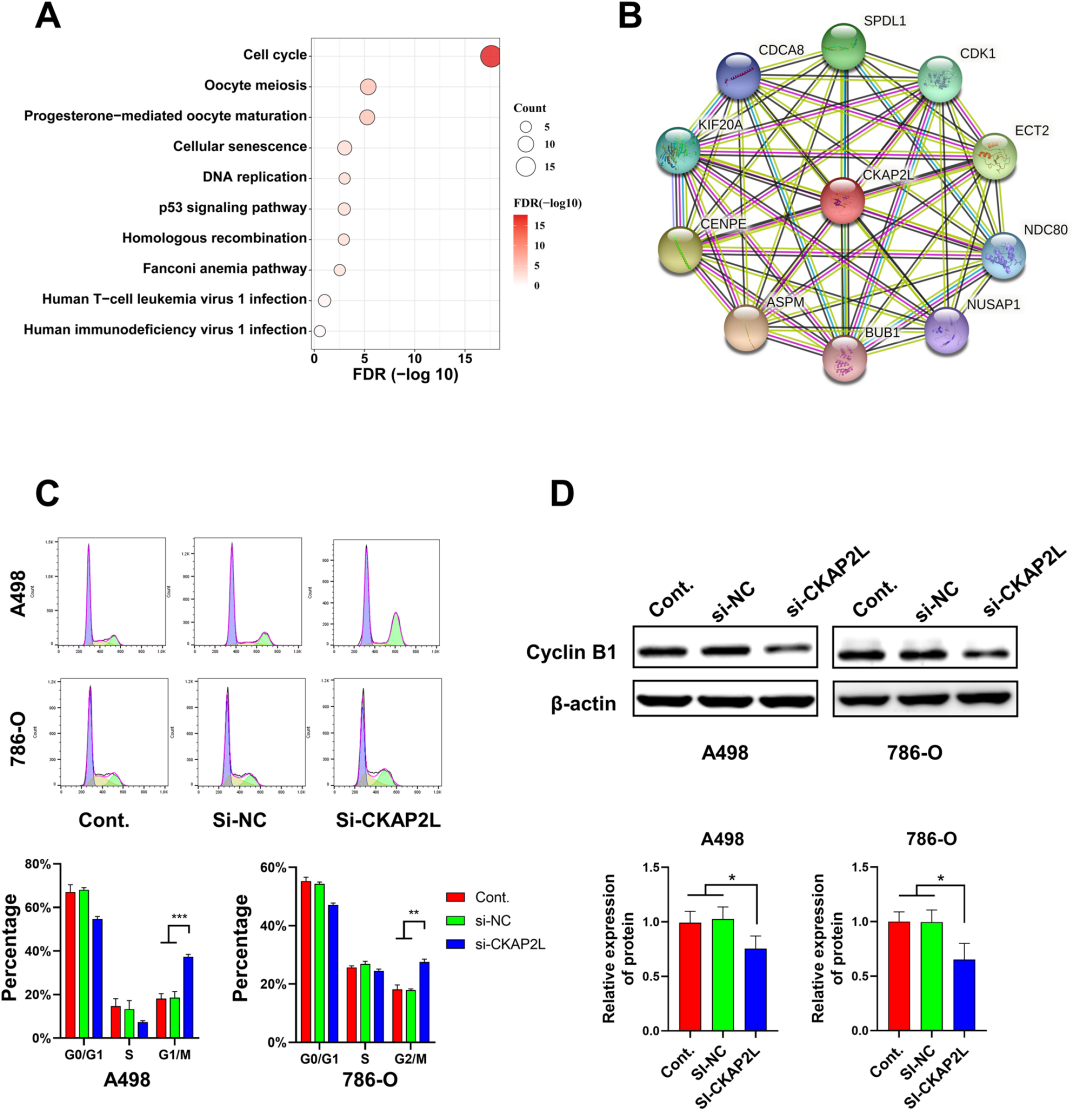
CKAP2L is involved in the regulation of the cell cycle (*p < 0.05; **p < 0.01; ***p < 0.001; ****p < 0.0001). (**A**) KEGG enrichment analysis results. (**B**) Protein–protein interaction network of CKAP2L. (**C**) Flow cytometry was performed to investigate the effect of CKAP2L knockdown on the cell cycle of A498 and 786-O cells. (**D**) Protein blotting demonstrated that the knockdown of CKAP2L resulted in decreased expression of cyclin B1 protein in A498 and 786-O cells. The uncropped original image can be obtained in Supplementary Fig. S8.

To reveal the mechanism by which CKAP2L influences the biological behavior of cancer cells, KEGG and GO analysis and a PPI network were conducted. The results of the KEGG analysis showed that CKAP2L is closely related to the cell cycle-related pathway (Fig. 7A). In GO analysis, CKAP2L was primarily involved in biological processes such as cell division, and mitotic sister chromatid segregation. Molecular functions were mainly related to microtubule binding and microtubule motor activity. Cellular components show a major distribution in the nucleoplasm. (Supplementary Fig. S4). The PPI network was displayed in Fig. 7B, and the proteins co-expressed with CKAP2L were mainly associated with the mitosis cycle. The specific functions of these proteins we summarized in Supplementary Table 1. Therefore, we hypothesize that CKAP2L may influence the biological behavior of cancer cells by regulating the cell cycle. Our analysis of the cell cycle phase distribution of siRNA-transfected cells revealed that CKAP2L knockdown markedly elevated the number of G2/M phase cells (Fig. 7C). In addition, Cyclin B1 was a key regulator of the G2/M cell cycle transition^13^. Knockdown of CKAP2L significantly decreased cyclin B1 protein in A498 cells and 786-O cells (Fig. 7D). The results of flow cytometry and western blot experiments verified our conjecture to a certain extent.

### Correlation between CKAP2L expression and tumor immune subtypes

**Figure 8:**
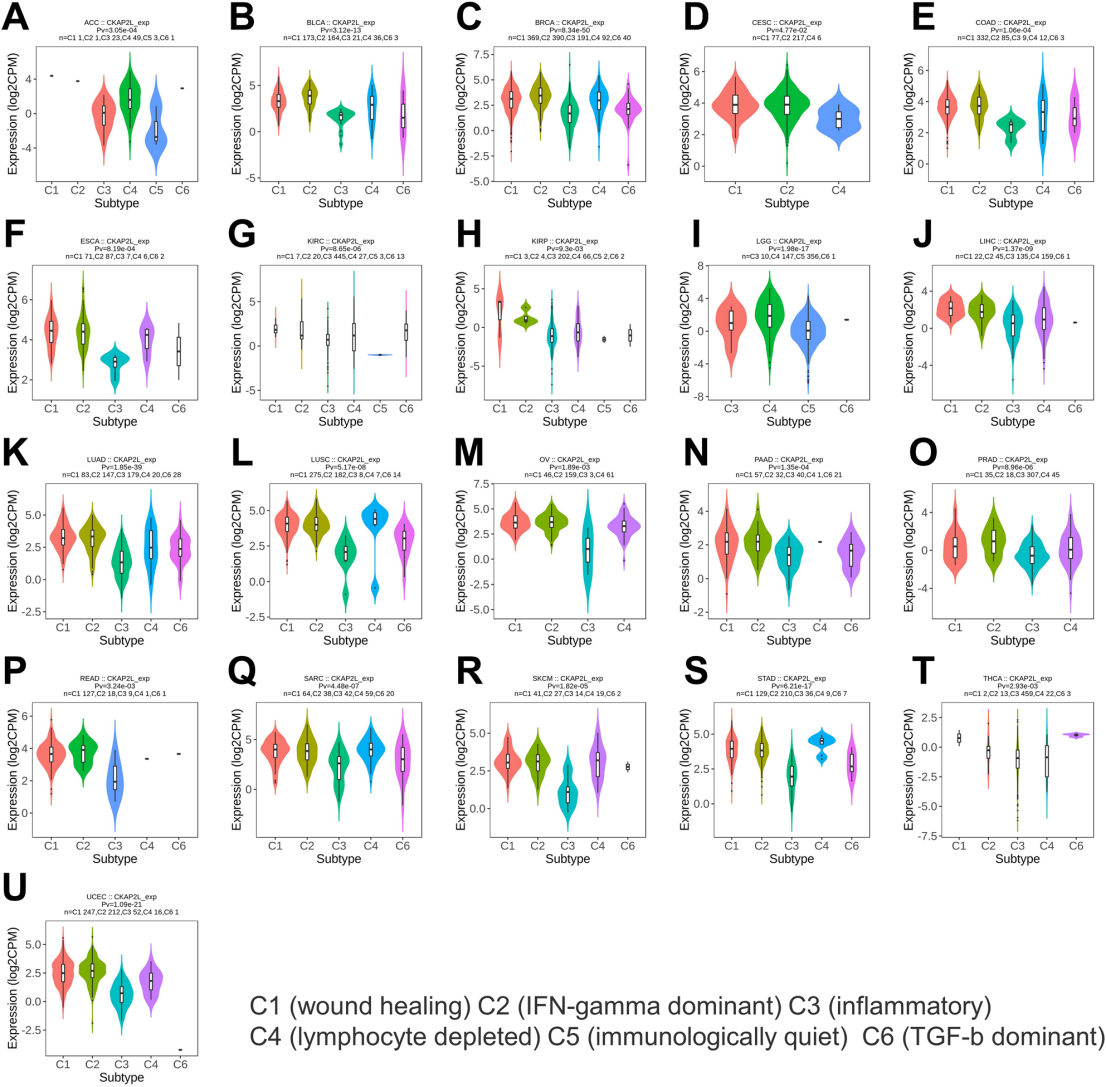
Correlation between CKAP2L expression and tumor immune subtypes. CKAP2L was closely associated with the immune subtypes of tumors in 21 tumors (these results were displayed with a p-value < 0.05).

In 2018, Thorsson et al. analyzed the immunogenomics of over 10,000 tumors to characterize six immune subtypes (Wound Healing, IFN-γ Dominant, Inflammatory, Lymphocyte Depleted, Immunologically Quiet and TGF-β Dominant) in 33 diverse cancers^14^. The different immune subtypes led to different immunotherapeutic effects. We examined CKAP2L expression in various tumor immune subtypes, as shown in Fig. 8A–U, CKAP2L was closely associated with the immune subtypes of tumors in 21 tumors.

### Association between CKAP2L expression and immune-related factors

**Figure 9:**
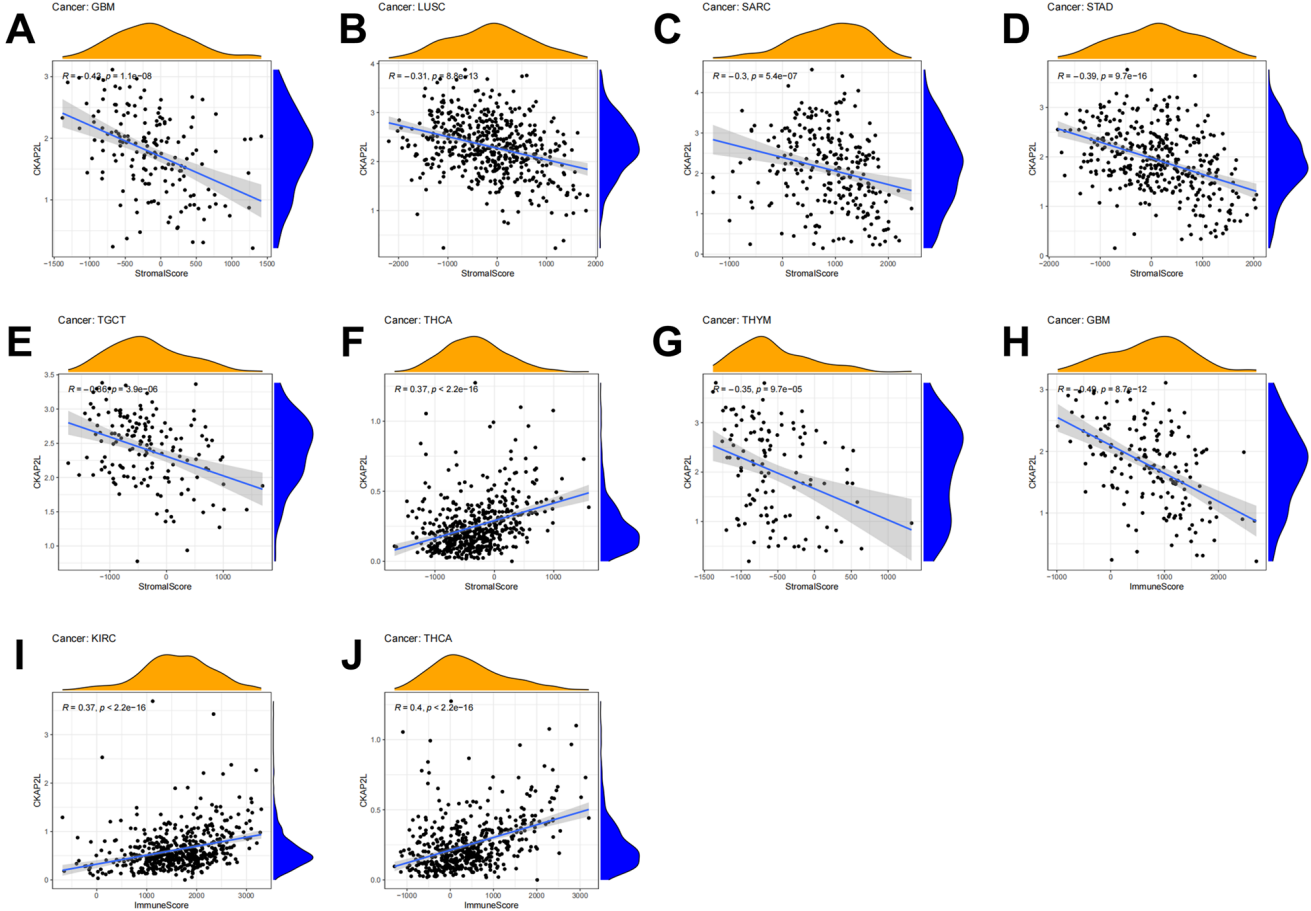
Correlations between CKAP2L expression and the ESTIMATE score. (The results were displayed with a p-value < 0.01 and |R| > 0.3). (**A**–**G**) Correlation between CKAP2L expression and stromal score in GMB (**A**), LUSC (**B**), SARC (**C**), STAD (**D**), TGCT (**E**), THCA (**F**), THYM (**G**). (**H**–**J**) Correlation between NOP2 expression and immune score in GBM (**H**), KIRC (**I**), and THCA (**J**).

**Figure 10:**
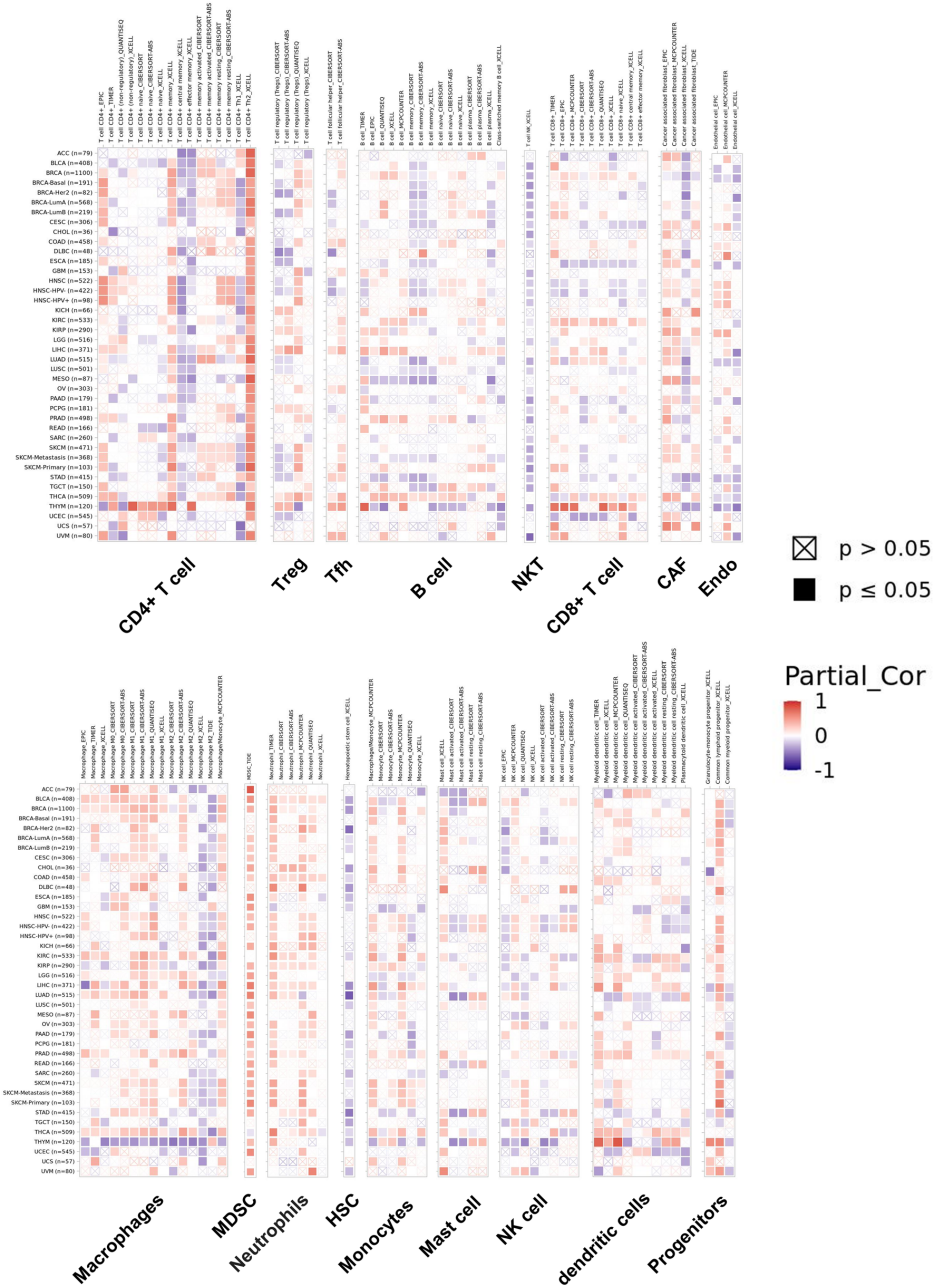
Correlation between CKAP2L expression and immune cell infiltration. Positive correlation in red and negative correlation in blue.

Since there were few studies of CKAP2L on tumor immunity, this paper comprehensively analyzed the correlation between CKAP2L expression and tumor immunity at the pan-cancer level. Figure 9A–J display the immune score and the stromal score (p-value < 0.01, |R| > 0.3). It is clear that the expression of CKAP2L was negatively related to the stromal score in GBM, LUSC, SARC, STAD, TGCT and THYM, but positively correlated in THCA. Additionally, the expression of CKAP2L was positively correlated with the immune score in KIRC and THCA, while it was negatively correlated in GBM. A summary of the relationship between CKAP2L expression and immune cell infiltration is shown in Fig. 10. The results revealed that the degree of immune cell infiltration was strongly related to the expression of CKAP2L in different tumors. For most types of tumors, CKAP2L expression was strongly positively related to Th2 cells, M1 macrophages, neutrophil and myeloid-derived suppressor cells (MDSC), and negatively correlated with Th1 cells and M2 macrophages.

**Figure 11:**
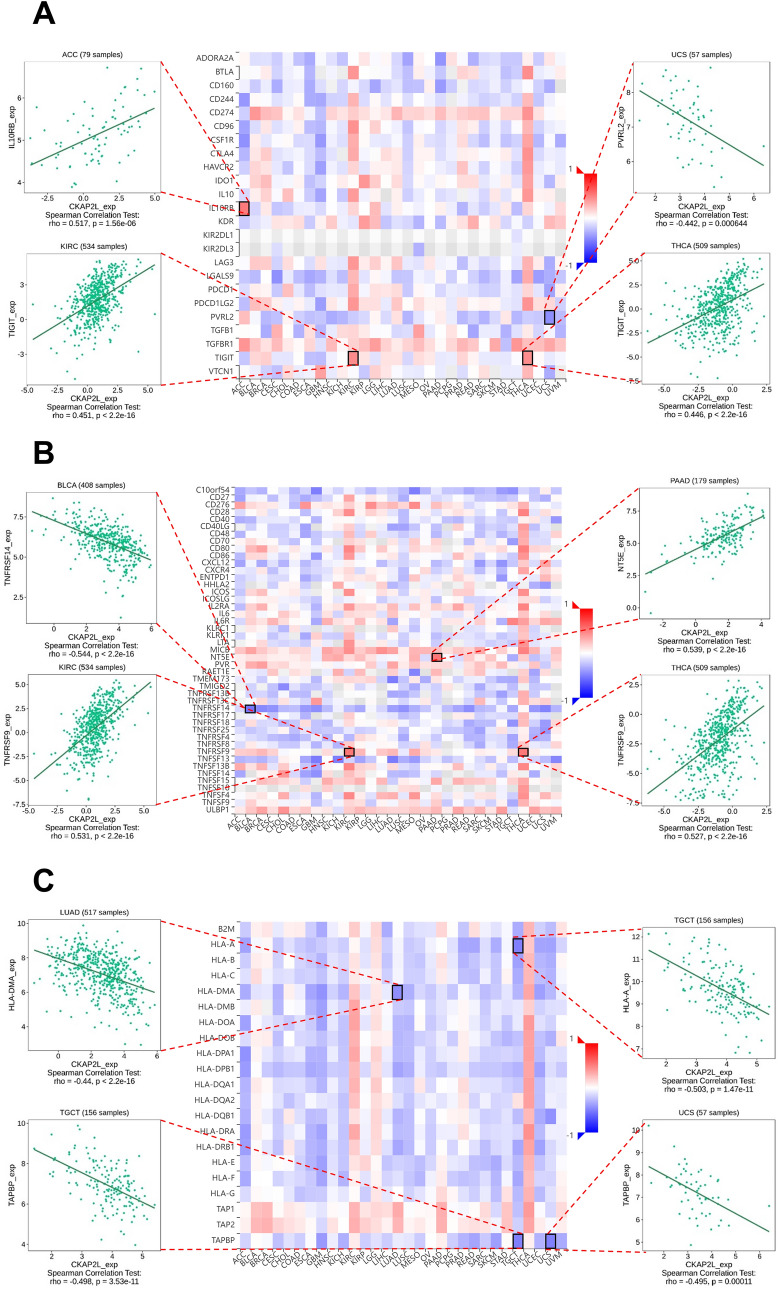
Correlations between CKAP2L expression and immunomodulators (blue represents a negative correlation, whereas red represents a positive correlation. Dot plots are used to show the top four strongest associations). (**A**) Correlation between CKAP2L expression and immune inhibitors. (**B**) Correlation between CKAP2L expression and immune stimulators. (**C**) Correlation between CKAP2L expression and MHC molecules.

Figure 11 summarizes the potential relationship between CKAP2L expression and immunomodulators, and we exhibit the top four most correlated for each immunomodulator by dot plots. For the 24 immune inhibitors (Fig. 11A), the expression of CKAP2L was positively related to IL10RB in ACC (R = 0.517), TIGIT in KIRC (R = 0.451), and TIGIT in THCA (R = 0.446), as well as negatively related to PVRL2 in UCS (R = − 0.442). Correlation analysis of immune stimulators (Fig. 11B) showed that CKAP2L expression was negatively correlated with TNFRSF14 in BLCA (R = − 0.544) and positively correlated with NT5E in PAAD (R = 0.539) and TNFRSF9 in both KIRC (R = 0.531) and THCA (R = 0.527). The relationship between CKAP2L and MHC molecules was also depicted in Fig. 11C, where there is a substantial negative correlation between CKAP2L and HLA-DMA in LUAD, TRPBP in UCS, as well as HLA-A and TRPBP in TGCT.

### Association between CKAP2L expression and immunotherapy

**Figure 12:**
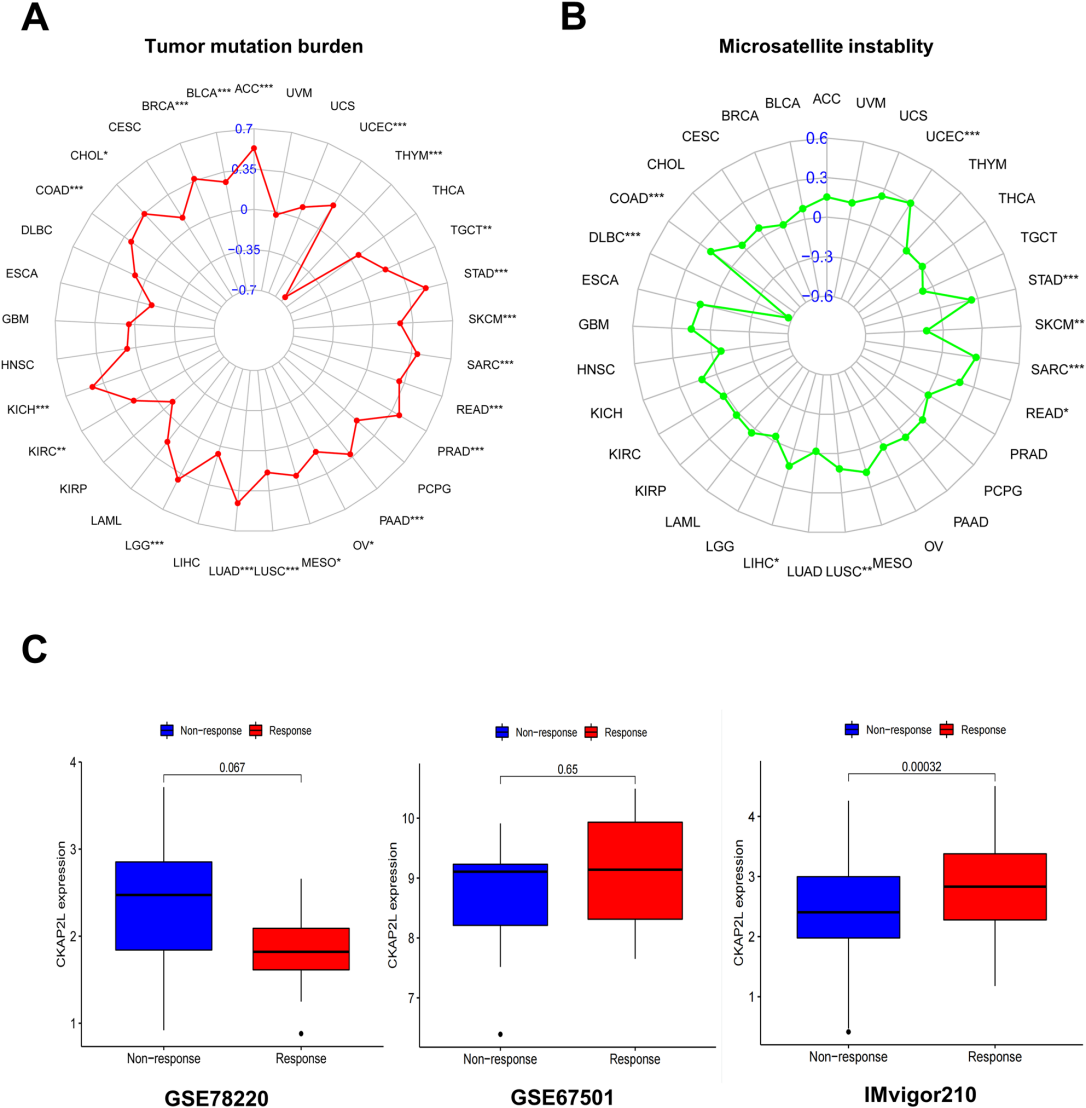
The correlation between CKAP2L and both immunotherapeutic markers and the immunotherapeutic response. (*p < 0.05; **p < 0.01; ***p < 0.001; ****p < 0.0001). (**A**) Correlations between CKAP2L expression and TMB. (**B**) Correlation between CKAP2L and MSI. (**C**) Correlations between CKAP2L and immunotherapeutic response in GSE78220, GES67501, and IMvigor210.

MSI, a pattern of hypermutation that affects genomic microsatellites, is brought on by flaws in the mismatch repair mechanism. TMB was defined as the total number of somatic gene coding mistakes, base substitutions, gene insertions, or deletions found in per million bases. Findings in some cancer types suggested that TMB and MSI were potential predictive biomarkers for immunotherapy^15,16^. As shown in the radar plot (Fig. 12A), the expression of CKAP2L was positively correlated with TMB in 15 type tumors, but negatively correlated in THYM. Regarding MSI (Fig. 12B), it had a positive correlation with the expression of CKAP2L in UCEC, STAD, SARC, READ, LUSC, LIHC and COAD, as well as a negative correlation in CKCM and DLBC. Additionally, we explored the connection between CKAP2L expression and immunotherapy response. As demonstrated in Fig. 12C, patients in the IMvigor210 cohort with higher CKAP2L expression levels were more responsive to immunotherapy. However, in the other two cohorts, CKAP2L expression does not differ between responder and non-responder groups.

## Discussion

CKAP2L is a mitotic spindle protein that has been previously shown to be overexpressed in some tumors and to be associated with tumor progression and patient prognosis. In prostate cancer, esophageal squamous cell carcinoma and glioblastoma, CKAP2L has been shown to affect the biological behavior of cancer cells by regulating the cell cycle^5–7^. A breast cancer study has shown that CKAP2L activates the AKT/mTOR signaling pathway and thus promotes breast cancer development^9^. In non-small cell lung cancer, CKAP2L directly interacted with RNA Pol II to contribute to the proliferation and growth of lung cancer in vitro and in vivo^15^. However, the relationship between CKAP2L and tumor progression and prognosis in other tumors remains unclear. Furthermore, a growing number of studies have revealed that the TME serves a critical role in tumor resistance, progression, invasion, and metastasis, significantly influencing the therapeutic effect and clinical outcome of malignant tumors^17–19^. Immunotherapy for tumors is also a popular research area today, offering new tools for the treatment of tumors. Because of the potential for saving lives demonstrated in clinical studies, “cancer immunotherapy” was named 2013’s Breakthrough of the Year by *Science*^20–22^. Nevertheless, the relationship between CKAP2L and tumor immune correlates is still not clearly articulated.

In this study, we comprehensively analyzed the expression of CKAP2L and its association with prognosis, chemotherapy and tumor immunity at the pan-cancer level by different databases, as well as partially validated our findings through in vitro experiments. Our results show that CKAP2L was overexpressed and in high activity in most of the 33 tumors and its expression increases with tumor stage in 7 cancer types, suggesting that it may be an oncogene in most tumors. Analysis of genetic alterations showed that CKAP2L had a 2.1% mutation rate in all tumors, with the highest rate of amplification and mutation, but the role of these alterations on CKAP2L is unclear. We speculate that may be the reason for high CKAP2L expression in tumors. Next, we used data from GEO to analyze the expression of CKAP2L in KIRC again and validated it at the tissue and cellular levels. Based on univariate Cox analysis and Kaplan–Meier survival curves, we found that CKAP2L is a marker of poor prognosis in many tumors.

CKAP2L knockdown in A498 and 786-O cells drastically decreased KIRC cells proliferation and migration, and invasive ability. To clarify the mechanism that CKAP2L affects the biological behavior of cells, the Enrichment analysis and PPI network were conducted. The result has shown that CKAP2L is primarily associated with cell cycle-related pathways and is involved in mitotic processes. Flow cytometry results showed that knockdown of CKAP2L resulted in cell cycle G2/M arrest. Similar to previous studies^5–7^, we suggested that CKAP2L may contribute to tumor progression in KIRC by influencing the biological behavior of cancer cells through the cell cycle too. However, we only validated the effect of CKAP2L in KIRC and further studies are needed to assess the role of CKAP2L in the other tumors.

TME consists of extracellular matrix, vascular network, lymphatic vessels, soluble molecules and cells. The majority of normal cells in tumor tissue are infiltrating stromal and immune cells, which not only interfere with the tumor signal in molecular studies but also play a crucial role in cancer biology^23–25^. The expression of CKAP2L was negatively correlated with the stromal score in GBM, LUSC, SARC, STAD, TGCT and THYM, but positively correlated in THCA. There is a positive correlation between CKAP2L expression and immune score in KIRC and THCA and a negative correlation in GBM.

This study also showed that the expression of CKAP2L and the level of infiltration of various immune cells were closely correlated in a variety of tumors. We found that CKAP2L expression linked negatively with Th1 cell infiltration and markedly positively with Th2 cell infiltration in 33 tumor types. Previous research has shown that Th2-polarized CD4+ T cells secrete cytokines such as IL-4, -5, -6 and -10, which reduce T cell antitumor activity and increase macrophage pro-tumor activity. Th1-polarized CD4+ cells, on the other hand, promote macrophage cytotoxicity by secreting cytokines such as TNF, which binds cytotoxic CD8+ T cells that have anti-tumor effects in vivo^26–28^. Furthermore, CKAP2L expression was positively associated with neutrophils and MDSC, they are both immune cells with pro-tumor activities^29–32^. These phenomena appear to explain why high CKAP2L expression is associated with a poor prognosis in most tumors. However, it is confusing that M1 macrophages, which are antitumor, are positively correlated with CKAP2L expression, and M2 macrophages, which have pro-tumor activity, are negatively correlated with CKAP2L expression^33^. This may be beneficial for the immunotherapy of tumors^34,35^, but further studies are needed to prove it.

The relationship between CKAP2L expression and three different immunomodulators was explored by the TISIDB database, and CKAP2L was positively correlated with most of the immunomodulators in KIRC and THCA. In addition, TGFβR1 in immune inhibitors, MICB and ULBP1 in immune stimulators were positively correlated with CKAP2L in most of the tumors. Current evidence suggests that TGFβR1 may influence disease progression by acting on stromal cells^36,37^. MICB and ULBP1, as NKG2D ligands, have been shown to be potential loci for targeted cancer therapy^38^.

Through the CellMiner database, we found that the sensitivity of most oncological chemotherapeutic drugs and CKAP2L expression were negatively correlated, suggesting that patients with elevated expression of CKAP2L may be insensitive to chemotherapeutic drugs. It has been reported that the sensitivity of chemotherapeutic agents in the patient is an important prognostic factor for their survival^39,40^, which may be a potential reason for the poor prognosis of patients with high CKAP2L expression.

The advent of immunotherapy has revolutionized cancer treatment, but its efficacy varies and only a subset of cancer patients can benefit from it^41^. TMB and MSI were potential predictive markers for immunotherapy^15,16,42^, CKAP2L was positively correlated with TMB and MSI in most type cancers, indicating that patients with elevated CKAP2L expression in these tumors responded better to immunotherapy.

This result was also verified in the IMvigor210 cohort study that patients with elevated expression of CKAP2L were better responded to immunotherapy. Interestingly, this result, in contrast to the traditional chemotherapeutic agents, implies that immunotherapy may address drug insensitivity in patients with high CKAP2L expression, and CKAP2L may serve as a potential biomarker to guide the choice of cancer treatment modality. In addition, there is growing evidence supporting the clinical value of combining appropriate doses of chemotherapy with immunotherapy^43,44^, and considering the particular relevance of CKAP2L to chemotherapy and immunotherapy, CKAP2L may provide novel ideas for the treatment of tumors against these targets.

In conclusion, CKAP2L can be used as a potential predictor of tumor prognosis and cancer therapy, providing immune-based antitumor strategies targeting CKAP2L.

## Materials and methods

### Data collection

Transcriptome data information data of various cancer types were obtained from the TCGA database. Expression data of CKAP2L in tumor tissues and normal tissues of the kidney were also obtained from the GEO database (GSE53757, GSE46699 and GSE66272). Data on protein expression detected by immunohistochemistry were downloaded from the Human Protein Atlas (HPA) database (https://www.proteinatlas.org/). The full names and abbreviations of 33 tumors were exhibited in Supplementary Table 2.

### Gene expression and genomic alterations analysis

The expression difference of CKAP2L between tumor tissues and normal tissues was analyzed by R studio (R version: 4.2.1) (http://www.r-project.org/). Furthermore, we analyzed TIMER2.0 data from different tumor tissues and normal tissues^45^. The activity of CKAP2L was compared by single sample gene set enrichment analysis (ssGSEA). The Cancer Cell Line Encyclopedia (CCLE) project has performed detailed genetic characterization of a large set of human cancer cell lines^46^. Therefore, we obtained CKAP2L expression data of various cancer cells from the CCLE database and visualized them by the ggplot2 package of R software. The cBioPortal platform (http://www.cbioportal.org) was used to analyze and visualize types and frequencies of genomic alterations of CKAP2L in tumors, 2922 samples from the TCGA database were involved.

### DNA methylation analysis of the CKAP2L in KIRC

DNA methylation can be used as a potential biomarker to predict patient prognosis. methSurv is a web-based tool for survival analysis based on CpG methylation patterns with 7358 methylation data from 25 different human cancers^47^. We used MethSurv to determine the expression and prognostic patterns of individual CpG methylation of CKAP2L in KIRC.

### Clinic correlation and survival prognosis

We analyzed the correlation between CKAP2L expression and tumor stage. Univariate Cox regression analysis was conducted by the R software to calculate the time-dependent prognostic value of CKAP2L in each cancer type. The survival curves of patients in each cancer type were created by the Kaplan–Meier (KM) method. In terms of survival results, there were four categories: overall survival (OS), disease-free survival (DFS), disease-specific survival (DSS), and progression-free survival (PFS). A statistical two-sided p-value < 0.05 was deemed significant.

### Analysis of the correlation between CKAP2L expression and drug response

The relationship between drug response and CKAP2L expression was analyzed by the CellMiner platform^48^, and the results were processed and visualized by R packages such as “impute”, “ggpubr” and “ggplot2” (displayed results with p-values greater than 0.05).

### Analysis of the correlation between CKAP2L expression and tumor immune subtypes

TISIDB (http://cis.hku.hk/TISIDB/index.php) is a website dedicated to the interaction of tumors and the immune system. We used it to explore the connection between the expression of CKAP2L and immune subtypes in human cancers.

### Analysis of the potential correlation between CKAP2L expression and immune-related factors

We analyzed immune and matrix components in the TME by the “estimate” package and demonstrated the relationship of CKAP2L with immune and matrix components utilizing ImmuneScore and StromalScore. We used the TIMER2.0 website to estimate the correlation between immune cell infiltration and CKAP2L expression. In addition, the TISIDB database was used to examine the potential connection between CKAP2L expression and immunomodulators. This study also explored the relationship of CKAP2L expression with TMB and MSI.

### Analysis of immunotherapeutic response

This study included and analyzed three immunotherapeutic groups (GSE78220, GES67501 and IMvigor210). In this study, patients were divided into responders and non-responders based on whether they achieved a complete response or partial response, or displayed symptoms of stable disease or progressing disease. Using Wilcoxon analysis, we determined the differences between responders and non-responders in CKAP2L expression.

### Construction of the protein–protein interaction network

A protein–protein interaction (PPI) network of CKAP2L including 10 related proteins was constructed using the STRING platform (https://string-db.org/).

### Enrichment analysis

We used the GEPIA2 database (http://gepia2.cancer-pku.cn/) to collect a total of 156 genes with a correlation coefficient greater than 0.6 with CKAP2L (Supplementary Table 3). KEGG and GO analyses were performed on these genes by the DAVID platform (https://david.ncifcrf.gov/). Finally, the results were visualized by the “ggrepel” and “ggplot2” R packages.

### Clinical specimens

In the Second Hospital of Tianjin Medical University, kidney cancer and adjacent tissue were obtained from patients undergoing surgery. Tissues were stored at − 80 °C after surgical excision. The study met the criteria approved by the Tianjin Medical University Health Medicine Research Ethics Committee.

### Cell lines and cell culture

Human kidney normal cells (HK-2) and cancer cell lines (Caki-1, 769-P, A498, and 786-O) were cultured in Dulbecco's modified Eagle's medium or RPMI 1640 medium.

### Cell siRNA transfection

The small interfering (si) RNA and Transfection reagent were produced by GenePharma company. The kidney cancer cells, A498 and 786-O, were transfected with CKAP2L siRNA or negative control (NC) siRNA. We display their sequence as follows:

- CKAP2L siRNA: GCUGAUGUCACAACCGUAATT and UUACGGUUGUGACAUCAGCTT.
- NC siRNA: UUCUUCGAACGUGUCACGUTT and ACGUGACAUGUUCGGAGAATT.

### Western blot analysis

Total proteins were extracted by using RIPA lysis buffer mixed. The same amounts of proteins were separated by gel electrophoresis and transferred to nitrocellulose membranes. Before hybridization with different antibodies, we cut the membrane according to the molecular weight of the target protein for subsequent experiments. We incubated the membranes overnight with different primary antibodies. After that, the transferred membranes were washed thrice for 15 min each time with TBST (TBS with Tween). Secondary antibodies were incubated with the membranes for 90 min. The following antibodies (Abs) were utilized: anti-CKAP2L Ab, anti-CyclinB1 Ab, and anti-β-Actin Ab. Western blot signals and images were obtained by BIO-RAD ChemiDoc XRS chemiluminescence system.

### Flow cytometry assay

We collected and fixed transfected cells with 70% ethanol and stained them according to the kit's instructions (Abbkine, Wuhan, China). Flow cytometric analysis was performed using the FACS Calibur flow cytometer. The cell fractions in the respective cell cycle phases were calculated by FLOWJO software (BD Biosciences, USA). Three repeats of each experiment were conducted.

### Cell counting Kit-8 (CCK-8) assay

Transfected cells were plated into 96-well with six repeats. Add CCK8 reagent at 1, 2, 3 and 4 days after cells were plated respectively. After 2 h incubation, absorbances at 450 nm were measured by a Spectra Max Plus Absorbance Microplate reader.

### Ethynyl-2-deoxyuridine (EdU) incorporation assay

Evaluation of kidney cancer cell proliferation through the EdU Cell Proliferation Image Kit (Abbkine, Wuhan, China). EdU is a thymidine nucleoside analog for DNA synthesis, which is added to cells during replication and detected by autoradiography. Transfected cells were seeded into a 10 cm confocal dish at a density of 8 × 10^4^ cells/dish. When the cells have grown to an appropriate density, stain the EdU and nuclear according to the instructions. Images were obtained by electron fluorescence microscopy (Nikon Eclipse 80i, Japan).

### Clone formation experiments

The transfected cells were seeded into 60 mm culture dishes in about 500 numbers. After two weeks of incubation, we used the 3.7% methanol and 0.1% crystal violet solution to fix and stain cells. The results were visualized and analyzed by ImageJ. Three repeats of each experiment were conducted.

### Transwell assay

To assess the cell's ability of invasion, the transwell test was performed. First, 100 μl of media (1% FBS) containing 5 × 10^4^ cells were seeded into the transwell insert that had been coated with Matrigel. The transfected cells were maintained in a low serum condition for 24 h. Put the transwell insert slightly into the lower chamber that contains 600 μl of 10% FBS medium. Following a 48 h incubation in 5% CO_2_ at 37 °C, the cells unpenetrated were removed by a swab, and the penetrated cells were fixed with 4% methanol. After washing with phosphate-buffered solution (PBS), the cells were stained with crystal violet solution. Three fields of view were obtained through the microscope irregularly. Images were analyzed by ImageJ. Three repeats of each experiment were conducted.

### Scratching wound healing assay

The transfected cells were plated in micro-insert 4-wells (ibidi GmbH, Germany). When the cell confluence reached 80–90%, remove the culture inserts to form a 500 μm wide scratch. The dishes were washed 3 times with PBS, and replaced with fresh medium without serum. The dishes were taken out for photography under a microscope (Olympus, Japan) at 0, 24 and 48 h after the scratch formed, respectively. Images were analyzed by ImageJ. Three repeats of each experiment were conducted.

### Statistical analysis

Comparison of gene expression in different tissues and cancer cell lines by the Wilcoxon rank-sum test or Student's *t* test. The correlation between CKAP2L expression and the patient prognosis was assessed by Kaplan–Meier analysis and univariate Cox regression analysis. The correlation of CKAP2L expression with targets such as immune cell infiltration, TMB, and MSI was assessed using the Spearman correlation test. The quantitative data from more than two experimental groups were analyzed by ANOVA to calculate the significance of the differences. Data were presented as means ± SD. A statistical two-sided p-value < 0.05 was deemed significant.

### Ethics declarations

All experiments were carried out with the Ethics Committee of the Second Hospital of Tianjin Medical University's approval, and before this study, informed consent was obtained from every patient. All procedures were carried out in conformity with the necessary standards and laws.

## Supplementary Information

Supplementary Information.

## Data Availability

The article/Supplementary Material contains the original contributions discussed in the study. If anyone needs data for this study they can contact the corresponding author at the following email address: drzhihong@126.com.
