# Supplementary material for: Pan-cancer analysis reveals the prognostic and immunotherapeutic value of cytoskeleton-associated protein 2-like
Source: Sci Rep. 2023 May 24;13:8368. doi: 10.1038/s41598-023-35633-3 (PMC10209194; doi:10.1038/s41598-023-35633-3)
Supplement: Supplementary file 1 — Supplementary Information. [file 41598_2023_35633_MOESM1_ESM.pdf]

Pan-cancer Analysis Reveals the Prognostic and Immunotherapeutic Value of Cytoskeleton-associated Protein 2-Like

Bocun Yi[1†], Qingfeng Fu[1†], Zhiwen Zheng[1†], Man Zhang[2], Dongze Liu[1], Zhengxin Liang[1], Shengxian XU[1], Zhihong Zhang[1*]

[1]Department of Urology, Tianjin Institute of Urology, The Second Hospital of Tianjin Medical University, Tianjin, China.
[2]Tianjin Key Laboratory of Metabolic Diseases, Tianjin Institute of Endocrinology, Chu Hsien-I Memorial Hospital of Tianjin Medical University, Tianjin, China.

***Correspondence:**

Zhihong Zhang

drzhihong@126.com

† These authors contributed equally to this work and share first authorship.

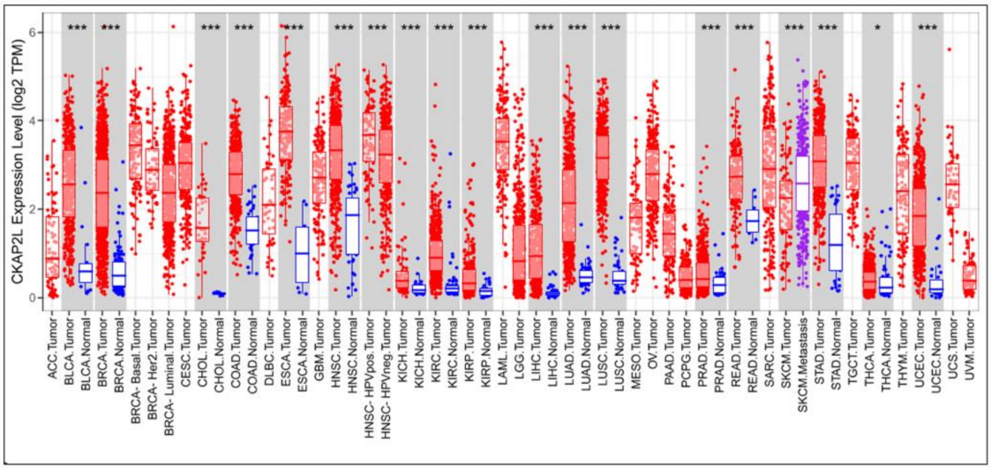

Supplementary figure S1. Expression of CKAP2L in various cancer types according to TIMER2.0. (*p < 0.05; **p < 0.01; and ***p < 0.001; ****p<0.0001.)

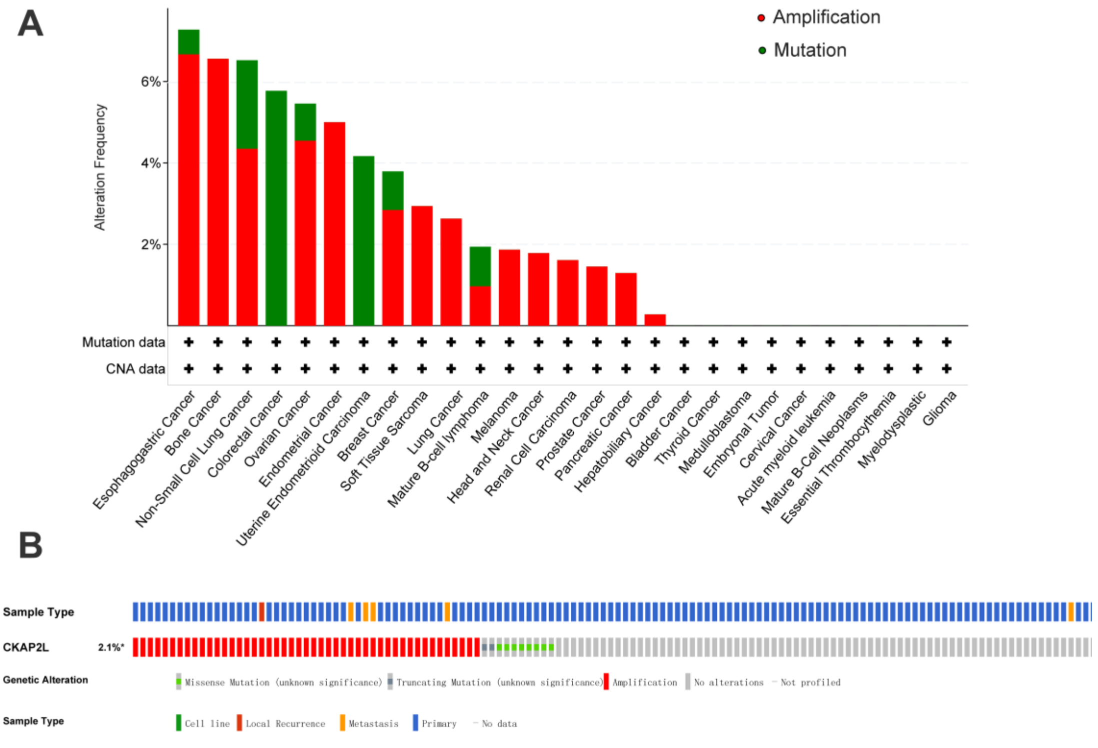

Supplementary figure S2. (A) Genomic alteration analysis of CKAP2L across cancer types. (B)General profile of genetic alterations in CKAP2L.

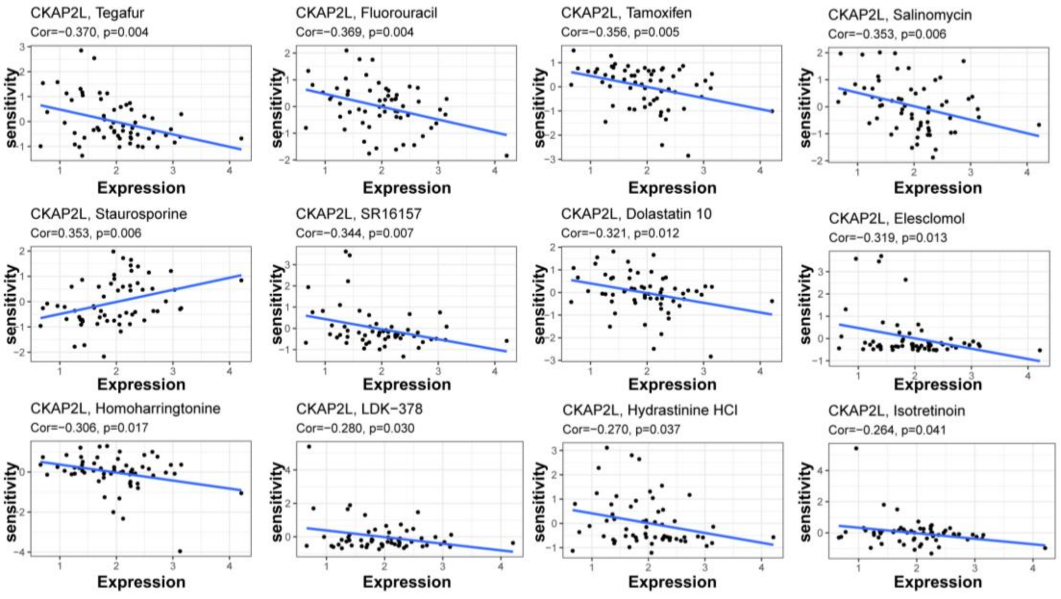

Supplementary figure S3. Relationship between CKAP2L expression and chemotherapy drug response.

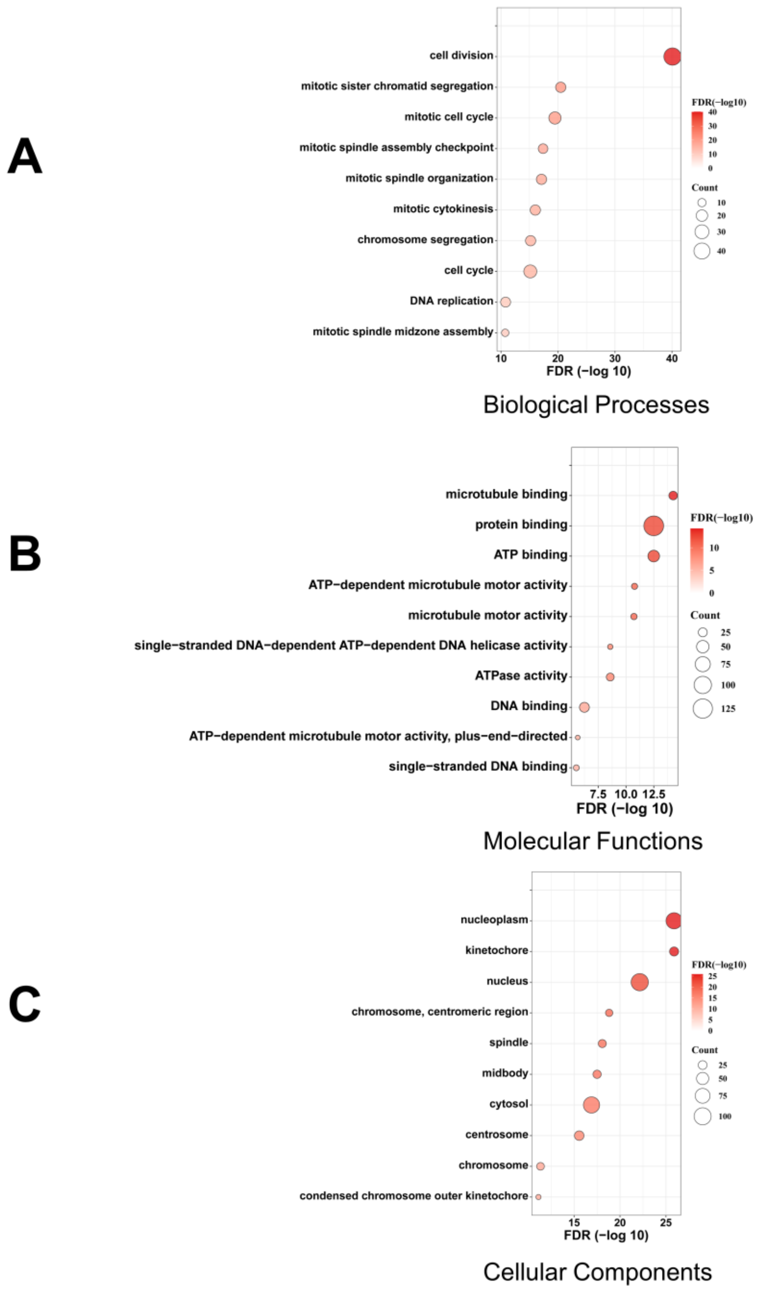

Supplementary figure S4. GO enrichment analysis of CKAP2L. (A) Biological processes. (B) Molecular Function. (C) Cellular Components.

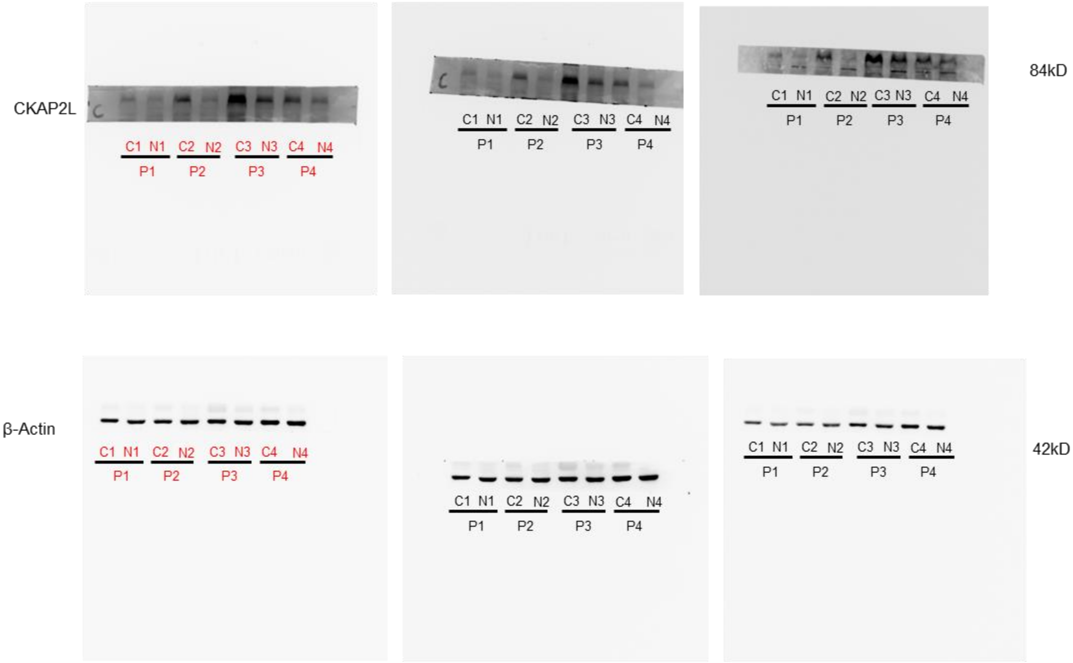

Supplementary figure S5. Original data of Figure 1G: CKAP2L expression levels in tumor tissues and adjacent tissues of four KIRC patients were measured using western-blot assays. Regarding the TMN staging of the four patients, P1 was stage T1, P2, P3 and P4 were stage T3. The red font marks the source of the cropped images in the manuscript. (In the course of the experiment, we cut the membranes according to the molecular weight of the protein before hybridization with the antibody)

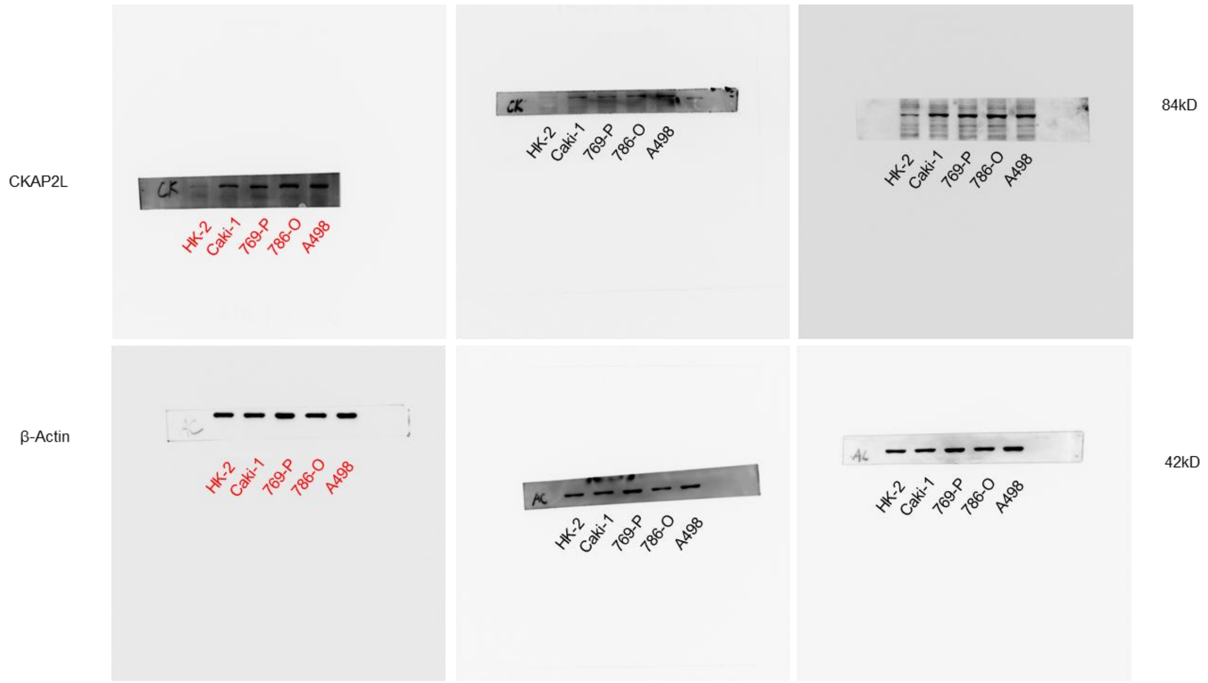

Supplementary figure S6. Original data of Figure 1H: Expression of CKAP2L in HK-2, Caki-1, 769-P, 786-O and A498 cells. The red font marks the source of the cropped images in the manuscript. (In the course of the experiment, we cut the membranes according to the molecular weight of the protein before hybridization with the antibody)

CKAP2L

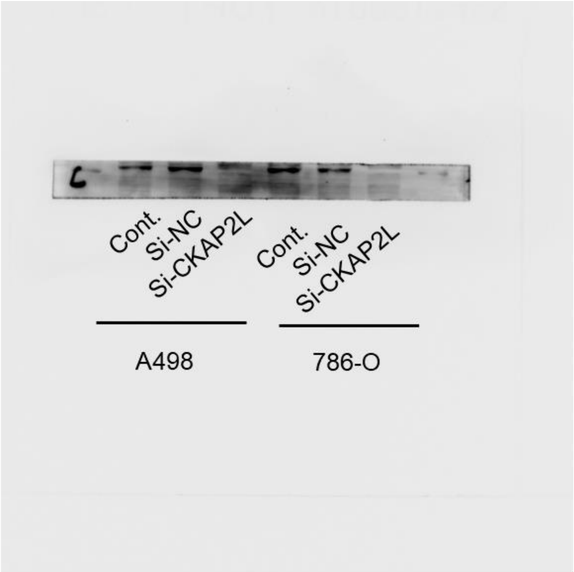

84kD

β-Actin

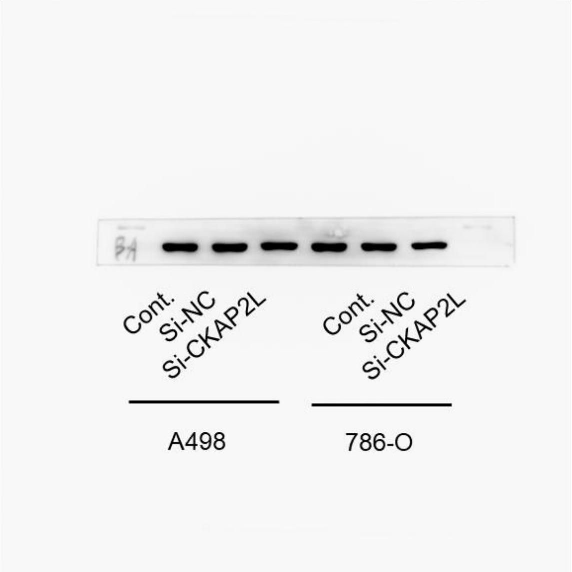

42kD

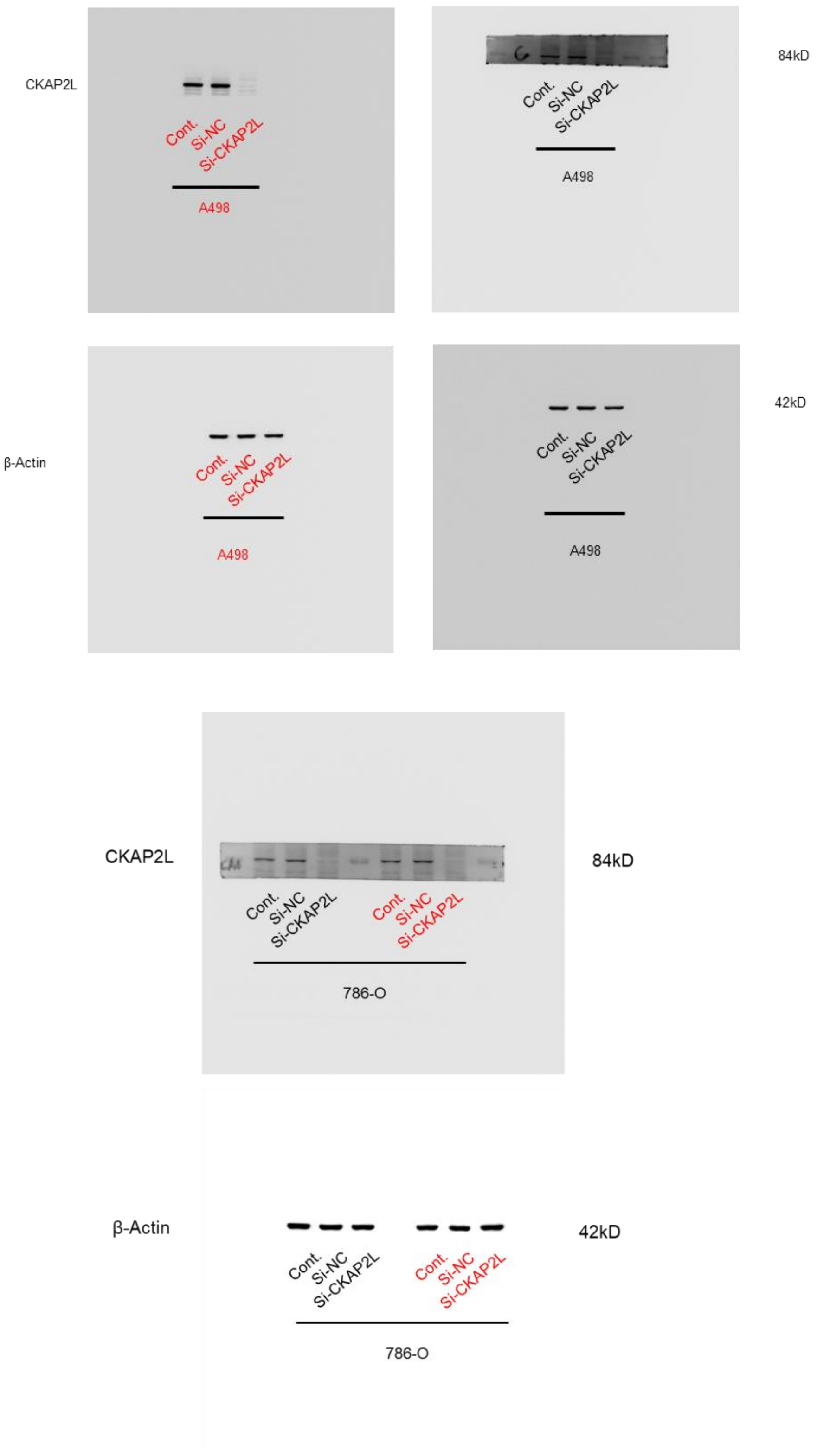

Supplementary figure S7. Original data of Figure 4A: Knockdown efficiency of CKAP2L after siRNA transfection of A498 and 786-O cells. The red font marks the source of the cropped images in the manuscript. (In the course of the experiment, we cut the membranes according to the molecular weight of the protein before hybridization with the antibody)

Cyclin B1

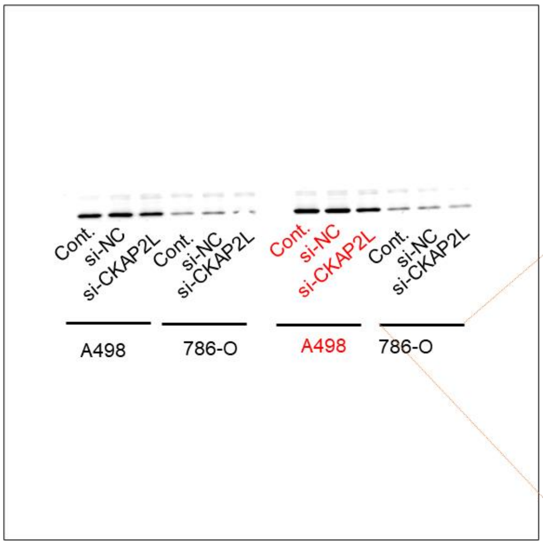

55kD

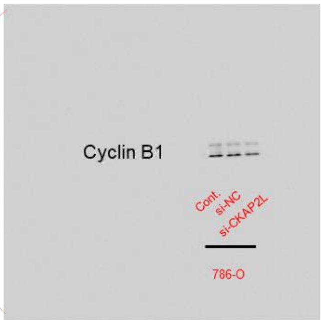

55kD

β-actin

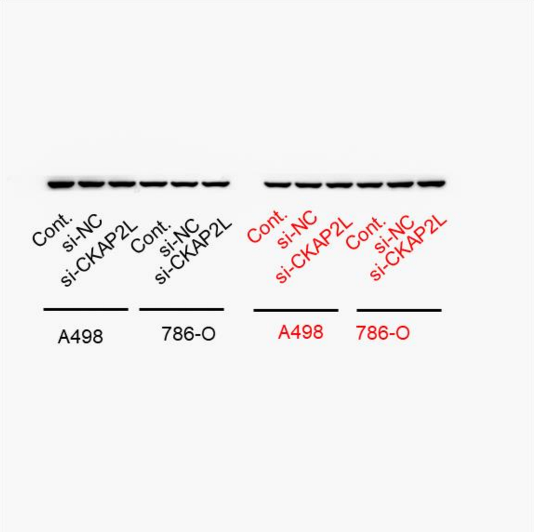

42kD

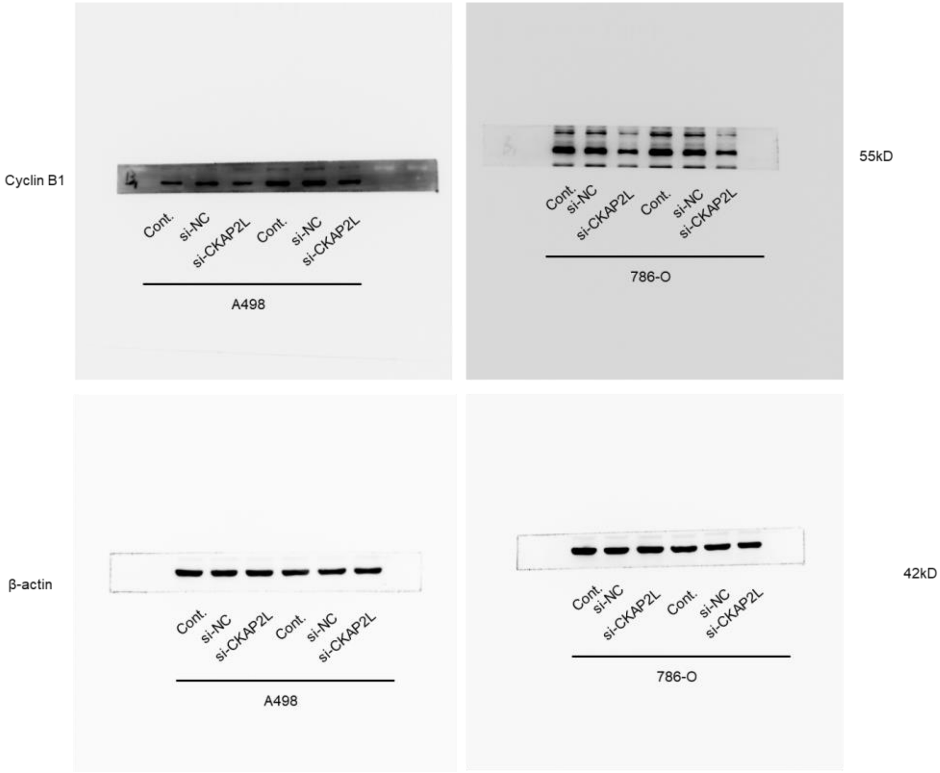

Supplementary figure S8. Original data of Figure 6D, the images in the red boxes are cropped and used in the manuscript.: Protein blotting demonstrated that the knockdown of CKAP2L resulted in decreased expression of cyclin B1 protein in A498 and 786-O cells. The red font marks the source of the cropped images in the manuscript. (In the course of the experiment, we cut the membranes according to the molecular weight of the protein before hybridization with the antibody)

Supplementary Table 1	
Name	domain_summary
ASPM	Abnormal spindle-like microcephaly-associated protein： Involved in mitotic spindle regulation and coordination of mitotic processes. The function in regulating microtubule dynamics at spindle poles including spindle orientation, astral microtubule density and poleward microtubule flux seems to depend on the association with the katanin complex formed by KATNA1 and KATNB1. Enhances the microtubule lattice severing activity of KATNA1 by recruiting the katanin complex to microtubules. Can block microtubule minus-end growth and reversely this function can be enhanced by the katanin complex [...]
BUB1	Mitotic checkpoint serine/threonine-protein kinase BUB1： Serine/threonine-protein kinase that performs 2 crucial functions during mitosis: it is essential for spindle-assembly checkpoint signaling and for correct chromosome alignment. Has a key role in the assembly of checkpoint proteins at the kinetochore, being required for the subsequent localization of CENPF, BUB1B, CENPE and MAD2L1. Required for the kinetochore localization of PLK1. Required for centromeric enrichment of AUKRB in prometaphase. Plays an important role in defining SGO1 localization and thereby affects sister chromat [...]
CDCA8	Cell division cycle associated 8; Borealin： Component of the chromosomal passenger complex (CPC), a complex that acts as a key regulator of mitosis. The CPC complex has essential functions at the centromere in ensuring correct chromosome alignment and segregation and is required for chromatin-induced microtubule stabilization and spindle assembly. Major effector of the TTK kinase in the control of attachment- error-correction and chromosome alignment
CDK1	Cyclin-dependent kinase 1： Plays a key role in the control of the eukaryotic cell cycle by modulating the centrosome cycle as well as mitotic onset; promotes G2-M transition, and regulates G1 progress and G1-S transition via association with multiple interphase cyclins. Required in higher cells for entry into S-phase and mitosis. Phosphorylates PARVA/actopaxin, APC, AMPH, APC, BARD1, Bcl-xL/BCL2L1, BRCA2, CALD1, CASP8, CDC7, CDC20, CDC25A, CDC25C, CC2D1A, CENPA, CSNK2 proteins/CKII, FZR1/CDH1, CDK7, CEBPB, CHAMP1, DMD/dystrophin, EEF1 proteins/EF-1, EZH2, KIF11/EG5, EGFR, FANCG, FOS, [...]
CENPE	Centromere-associated protein E： Microtubule plus-end-directed kinetochore motor which plays an important role in chromosome congression, microtubule- kinetochore conjugation and spindle assembly checkpoint activation. Drives chromosome congression (alignment of chromosomes at the spindle equator resulting in the formation of the metaphase plate) by mediating the lateral sliding of polar chromosomes along spindle microtubules towards the spindle equator and by aiding the establishment and maintenance of connections between kinetochores and spindle microtubules. The transport of pole-pr [...]
CKAP2L	Cytoskeleton-associated protein 2-like： Microtubule-associated protein required for mitotic spindle formation and cell-cycle progression in neural progenitor cells
ECT2	Protein ECT2： Guanine nucleotide exchange factor (GEF) that catalyzes the exchange of GDP for GTP. Promotes guanine nucleotide exchange on the Rho family members of small GTPases, like RHOA, RHOC, RAC1 and CDC42. Required for signal transduction pathways involved in the regulation of cytokinesis. Component of the centralspindlin complex that serves as a microtubule-dependent and Rho-mediated signaling required for the myosin contractile ring formation during the cell cycle cytokinesis. Regulates the translocation of RHOA from the central spindle to the equatorial region. Plays a role i [...]
KIF20A	Kinesin-like protein KIF20A;： Mitotic kinesin required for chromosome passenger complex (CPC)-mediated cytokinesis. Following phosphorylation by PLK1, involved in recruitment of PLK1 to the central spindle. Interacts with guanosine triphosphate (GTP)-bound forms of RAB6A and RAB6B. May act as a motor required for the retrograde RAB6 regulated transport of Golgi membranes and associated vesicles along microtubules. Has a microtubule plus end-directed motility; Kinesins
NDC80	Kinetochore protein NDC80 homolog;： Acts as a component of the essential kinetochore- associated NDC80 complex, which is required for chromosome segregation and spindle checkpoint activity. Required for kinetochore integrity and the organization of stable microtubule binding sites in the outer plate of the kinetochore. The NDC80 complex synergistically enhances the affinity of the SKA1 complex for microtubules and may allow the NDC80 complex to track depolymerizing microtubules. Plays a role in chromosome congression and is essential for the end-on attachment of the kinetochores to spin [...]
NUSAP1	Nucleolar and spindle-associated protein 1： Microtubule-associated protein with the capacity to bundle and stabilize microtubules (By similarity). May associate with chromosomes and promote the organization of mitotic spindle microtubules around them; Belongs to the NUSAP family
SPDL1	Spindle apparatus coiled-coil protein 1;： Protein Spindly; Required for the localization of dynein and dynactin to the mitotic kintochore. Dynein is believed to control the initial lateral interaction between the kinetochore and spindle microtubules and to facilitate the subsequent formation of end-on kinetochore-microtubule attachments mediated by the NDC80 complex. Also required for correct spindle orientation. Does not appear to be required for the removal of spindle assembly checkpoint (SAC) proteins from the kinetochore upon bipolar spindle attachment. Acts as an adapter protein li [...]

Supplementary table 1. Specific functions of various proteins in PPI analysis.

Supplementary Table 2

Abbreviations and details of the 33 cancer types in this study.

ACC	Adrenocortical carcinoma
BLCA	Bladder Urothelial Carcinoma
BRCA	Breast invasive carcinoma
CESC	Cervical squamous cell carcinoma and endocervical [illegible]
CHOL	Cholangiocarcinoma
COAD	Colon adenocarcinoma
DLBC	Lymphoid Neoplasm Diffuse Large B-cell Lymphoma
ESCA	Esophageal carcinoma
GBM	Glioblastoma multiforme
HNSC	Head and Neck squamous cell carcinoma
KICH	Kidney Chromophobe
KIRC	Kidney renal clear cell carcinoma
KIRP	Kidney renal papillary cell carcinoma
LAML	Acute Myeloid Leukemia
LGG	Brain Lower Grade Glioma
LIHC	Liver hepatocellular carcinoma
LUAD	Lung adenocarcinoma
LUSC	Lung squamous cell carcinoma
MESO	Mesothelioma
OV	Ovarian serous cystadenocarcinoma
PAAD	Pancreatic adenocarcinoma
PCPG	Pheochromocytoma and Paraganglioma
PRAD	Prostate adenocarcinoma
READ	Rectum adenocarcinoma
SARC	Sarcoma
SKCM	Skin Cutaneous Melanoma
STAD	Stomach adenocarcinoma
TGCT	Testicular Germ Cell Tumors
THCA	Thyroid carcinoma
THYM	Thymoma
UCEC	Uterine Corpus Endometrial Carcinoma
UCS	Uterine Carcinosarcoma
UVM	Uveal Melanoma

Supplementary table 2. The full names and abbreviations of 33 tumors.

Supplementary Table 3		
Gene Symbol	Gene ID	PCC
BUB1	ENSG00000169679.14	0.83
KIF14	ENSG00000118193.11	0.8
RACGAP1	ENSG00000161800.12	0.8
KIF11	ENSG00000138160.5	0.79
KIF4A	ENSG00000090889.11	0.79
ARHGAP11A	ENSG00000198826.10	0.79
SGOL2	ENSG00000163535.17	0.79
CENPI	ENSG00000102384.13	0.78
KIF23	ENSG00000137807.13	0.78
TPX2	ENSG00000088325.15	0.78
NCAPG	ENSG00000109805.9	0.77
NCAPH	ENSG00000121152.9	0.77
DEPDC1	ENSG00000024526.16	0.76
CCNA2	ENSG00000145386.9	0.76
MKI67	ENSG00000148773.12	0.76
CENPE	ENSG00000138778.11	0.76
KIF18A	ENSG00000121621.6	0.75
DLGAP5	ENSG00000126787.12	0.75
NUSAP1	ENSG00000137804.12	0.75
KIF2C	ENSG00000142945.12	0.74
CEP55	ENSG00000138180.15	0.74
ASPM	ENSG00000066279.16	0.74
RRM2	ENSG00000171848.13	0.74
PLK4	ENSG00000142731.10	0.74
PRC1	ENSG00000198901.13	0.74
CLSPN	ENSG00000092853.13	0.73
FANCI	ENSG00000140525.17	0.73
KIF18B	ENSG00000186185.13	0.73
MELK	ENSG00000165304.7	0.73
TTK	ENSG00000112742.9	0.73
KIAA1524	ENSG00000163507.13	0.73
NCAPG2	ENSG00000146918.19	0.73
SGOL1	ENSG00000129810.14	0.73
GTSE1	ENSG00000075218.18	0.73
PLK1	ENSG00000166851.14	0.72
HMMR	ENSG00000072571.19	0.72
HJURP	ENSG00000123485.11	0.72
KIF20A	ENSG00000112984.11	0.72
ZWILCH	ENSG00000174442.11	0.72
SKA1	ENSG00000154839.9	0.72
CENPL	ENSG00000120334.15	0.72
CKAP2	ENSG00000136108.14	0.72
CCNB2	ENSG00000157456.7	0.72
CDCA5	ENSG00000146670.9	0.71
CENPO	ENSG00000138092.10	0.71
INCENP	ENSG00000149503.12	0.71
KIF20B	ENSG00000138182.14	0.71
KIFC1	ENSG00000237649.7	0.7
LMNB2	ENSG00000176619.10	0.7
GSG2	ENSG00000177602.5	0.69
CDK1	ENSG00000170312.15	0.69
MCM10	ENSG00000065328.16	0.69
NDC80	ENSG00000080986.12	0.69
DTL	ENSG00000143476.17	0.69
STIL	ENSG00000123473.15	0.69
MAD2L1	ENSG00000164109.13	0.69
FOXM1	ENSG00000111206.12	0.68
CHEK1	ENSG00000149554.12	0.68
NUF2	ENSG00000143228.12	0.68
SMC2	ENSG00000136824.18	0.68
CENPF	ENSG00000117724.12	0.68
SHCBP1	ENSG00000171241.8	0.68
CCNB1	ENSG00000134057.14	0.68
FBXO5	ENSG00000112029.9	0.68
GINS1	ENSG00000101003.9	0.68
MCM4	ENSG00000104738.16	0.68
DBF4	ENSG00000006634.7	0.68
DDIAS	ENSG00000165490.12	0.67
SPDL1	ENSG00000040275.16	0.67
DIAPH3	ENSG00000139734.17	0.67
MCM6	ENSG00000076003.4	0.67
MMS22L	ENSG00000146263.11	0.67
CASC5	ENSG00000137812.19	0.67
ESCO2	ENSG00000171320.14	0.67
POLQ	ENSG00000051341.13	0.67
TMPO	ENSG00000120802.13	0.67
SPC25	ENSG00000152253.8	0.67
MCM8	ENSG00000125885.13	0.67
ATAD2	ENSG00000156802.12	0.66
AURKA	ENSG00000087586.17	0.66
RRM1	ENSG00000167325.14	0.66
LMNB1	ENSG00000113368.11	0.66
MASTL	ENSG00000120539.14	0.66
CDKN3	ENSG00000100526.19	0.66
E2F7	ENSG00000165891.15	0.66
BIRC5	ENSG00000089685.14	0.66
MTBP	ENSG00000172167.7	0.66
EXO1	ENSG00000174371.16	0.66
SASS6	ENSG00000156876.9	0.66
NCAPD2	ENSG00000010292.12	0.66
CDC25C	ENSG00000158402.18	0.66
CCNF	ENSG00000162063.12	0.65
BUB1B	ENSG00000156970.12	0.65
NEK2	ENSG00000117650.12	0.65
ANLN	ENSG00000011426.10	0.65
BRIP1	ENSG00000136492.8	0.65
ESPL1	ENSG00000135476.11	0.65
BRCA1	ENSG00000012048.19	0.65
SMC4	ENSG00000113810.15	0.64
MTFR2	ENSG00000146410.11	0.64
POLR2D	ENSG00000144231.10	0.64
ZWINT	ENSG00000122952.16	0.64
CENPA	ENSG00000115163.14	0.64
CDCA8	ENSG00000134690.10	0.64
PARPBP	ENSG00000185480.11	0.64
KPNA2	ENSG00000182481.8	0.64
FAM72B	ENSG00000188610.12	0.64
FANCB	ENSG00000181544.13	0.64
KIF15	ENSG00000163808.16	0.64
WDR76	ENSG00000092470.11	0.64
CENPK	ENSG00000123219.12	0.64
RBL1	ENSG00000080839.11	0.64
TICRR	ENSG00000140534.13	0.63
NEMP1	ENSG00000166881.9	0.63
FAM72D	ENSG00000215784.5	0.63
ECT2	ENSG00000114346.13	0.63
IQGAP3	ENSG00000183856.10	0.63
RAD51AP1	ENSG00000111247.14	0.63
GMPS	ENSG00000163655.15	0.63
OIP5	ENSG00000104147.8	0.63
NUP155	ENSG00000113569.15	0.63
FIGNL1	ENSG00000132436.11	0.63
C1orf112	ENSG00000000460.16	0.62
PBK	ENSG00000168078.9	0.62
XRCC2	ENSG00000196584.2	0.62
SKA3	ENSG00000165480.15	0.62
LIN9	ENSG00000183814.15	0.62
BORA	ENSG00000136122.15	0.62
CEP78	ENSG00000148019.12	0.62
TOPBP1	ENSG00000163781.12	0.62
CDCA4	ENSG00000170779.10	0.62
ACTR3	ENSG00000115091.11	0.62
LRR1	ENSG00000165501.16	0.62
EZH2	ENSG00000106462.10	0.62
MCM2	ENSG00000073111.13	0.62
NDC1	ENSG00000058804.11	0.62
DSCC1	ENSG00000136982.5	0.62
C17orf53	ENSG00000125319.14	0.61
ORC6	ENSG00000091651.8	0.61
TTF2	ENSG00000116830.11	0.61
FEN1	ENSG00000168496.3	0.61
TIMELESS	ENSG00000111602.11	0.61
RAD51	ENSG00000051180.16	0.61
PRPF40A	ENSG00000196504.15	0.61
ORC1	ENSG00000085840.12	0.61
FAM72C	ENSG00000263513.5	0.61
RFC5	ENSG00000111445.13	0.61
RQCD1	ENSG00000144580.13	0.61
TBC1D31	ENSG00000156787.16	0.61
NDE1	ENSG00000072864.12	0.61
DBF4P1	ENSG00000235489.4	0.61
DEK	ENSG00000124795.14	0.61
RFWD3	ENSG00000168411.13	0.61
DCLRE1B	ENSG00000118655.4	0.61
SRSF3	ENSG00000112081.16	0.61
AURKB	ENSG00000178999.12	0.61

Supplementary table 3. The 156 genes were obtained from the GEPIA2 database with a correlation coefficient greater than 0.6 with CKAP2L.
